# Enantiomeric Fractions Reveal Differences in the Atropselective Disposition of 2,2′,3,5′,6-Pentachlorobiphenyl (PCB 95) in Wildtype, *Cyp2abfgs*-Null, and CYP2A6-Humanized Mice

**DOI:** 10.1021/acs.chemrestox.3c00128

**Published:** 2023-07-19

**Authors:** Xueshu Li, Amanda J. Bullert, Weiguo Han, Weizhu Yang, Qing-Yu Zhang, Xinxin Ding, Hans-Joachim Lehmler

**Affiliations:** † Department of Occupational and Environmental Health, College of Public Health, University of Iowa, Iowa City, Iowa 52242, United States; ‡ Interdisciplinary Graduate Program in Neuroscience, University of Iowa, Iowa City, Iowa 52242, United States; § Department of Pharmacology and Toxicology, College of Pharmacy, University of Arizona, Tucson, Arizona 85721, United States

## Abstract

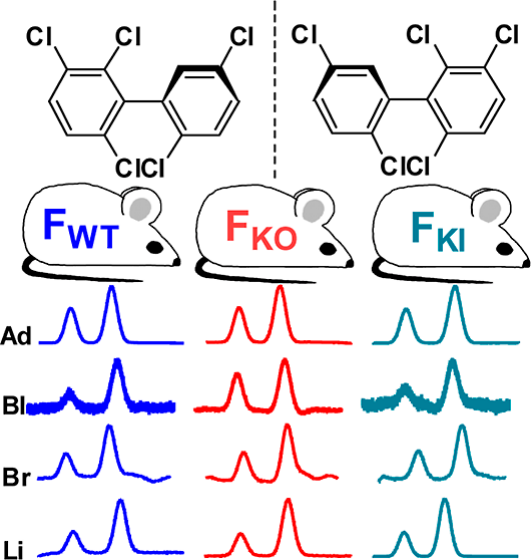

Polychlorinated biphenyls (PCBs) are environmental contaminants that can cause neurotoxicity. PCBs, such as PCB 95 (2,2′,3,5′,6-pentachlorobiphenyl), can be metabolized by cytochrome P450 enzymes into neurotoxic metabolites. To better understand how the metabolism of PCB 95 affects neurotoxic outcomes, we conducted a study on the disposition of PCB 95 in transgenic mouse models. The mice were given a single oral dose of PCB 95 (1.0 mg/kg) and were euthanized 24 h later for analysis. PCB 95 levels were highest in adipose tissue, followed by the liver, brain, and blood. Adipose tissue levels were significantly higher in wild-type (WT) mice than in Cyp2abfgs-null (KO) or CYP2A6-transgenic (KI) mice. We also observed genotype-dependent differences in the enrichment of aS-PCB 95 in female mice, with a less pronounced enrichment in KO than WT and KI mice. Ten hydroxylated PCB 95 metabolites were detected in blood and tissue across all exposure groups. The metabolite profiles differed across tissues, while sex and genotype-dependent differences were less pronounced. Total OH-PCB levels were highest in the blood, followed by the liver, adipose tissue, and brain. Total OH-PCB blood levels were lower in KO than in WT mice, while the opposite trend was observed in the liver. In male mice, total OH-PCB metabolite levels were significantly lower in KI than in WT mice in blood and the liver, while the opposite trend was observed in female mice. In conclusion, the study highlights the differences in the atropselective disposition of PCB 95 and its metabolites in different types of mice, demonstrating the usefulness of these transgenic mouse models for characterizing the role of PCB metabolism in PCB neurotoxicity.

## Introduction

Polychlorinated biphenyls (PCBs) are a class of organic compounds that were manufactured for diverse industrial and commercial applications, such as transformers and capacitors.^1^ PCBs persist in the environment where they bioaccumulate and biomagnify in aquatic and terrestrial food chains, posing a risk to wildlife and human health. Their production has been banned worldwide, and their use is being phased out under the Stockholm Convention on Persistent Organic Pollutants. However, some PCB congeners continue to be produced as byproducts of industrial processes, for example, the production of paint pigments^2^ and silicone rubber,^3^ resulting in current environmental and occupational PCB exposures.^4^ Humans are primarily exposed to PCBs via diet or inhalation.^5,6^ PCBs have been implicated in adverse cancer and non-cancer health outcomes.^7^ Importantly, PCB congeners with multiple *ortho* chlorine substituents, such as PCB 95, are implicated in PCB developmental neurotoxicity.^8,9^

PCB 95 is a constituent of technical PCB mixtures^10^ and has been detected in human postmortem brain tissue.^11,12^ It is one of 19 PCB congeners that display axial chirality, i.e., it exists as two rotational isomers, or atropisomers, that are non-superimposable mirror images of each other.^13^ PCB 95 is a potent sensitizer of the ryanodine receptor (RyR)^14^ that affects dendritic architecture.^15^ Moreover, pure atropisomers of PCB 95 and other chiral PCB congeners display stereoselectivity toward RyRs and developing neuronal networks.^16−18^ Exposure of rats to PCB 95 via the maternal diet promotes dendritic growth by a mechanism involving RyRs^19^ and alters behavior.^20^ In vitro and in vivo metabolism studies demonstrate that PCB 95 is atropselectively oxidized by CYP2 enzymes, such as human CYP2A6 and CYP2B6, to hydroxylated metabolites in rats and other mammals.^21−26^ These metabolites are present in the brain of mice exposed developmentally via the maternal diet^24^ and are also RyR active.^27^ These findings suggest that atropselective metabolism by specific cytochrome P450 enzymes may play an overlooked role in the developmental neurotoxicity of RyR-active PCBs, such as PCB 95.

Although in vitro studies provide mechanistic insights into the neurotoxicity of PCB metabolites, in vivo studies are needed to characterize how cytochrome P450 enzyme-mediated metabolism affects neurotoxic outcomes following developmental PCB exposure. Transgenic mouse models are a valuable tool to determine the role of hepatic metabolism in the toxicity of PCBs. For example, deleting the steroid and xenobiotic receptor (SXR), a nuclear transcription factor regulating PCB metabolism, affects PCB metabolite levels and toxic outcomes in mice.^28^ Similarly, the disposition of PCB 91 (2,2′,3,4′,6-pentachlorobiphenyl) and PCB 136 (2,2′,3,3′,6,6′-hexachlorobipheny), two PCB congeners structurally related to PCB 95, has been characterized in a mouse model with a liver-specific deletion of cytochrome P450 reductase, the obligate electron donor of cytochrome P450 enzymes.^29−31^ Genetic differences in CYP1A and CYP1B enzymes alter the toxicokinetics of PCBs and affect PCB developmental neurotoxicity.^32,33^ Finally, maternal CYP1A2 levels in the liver correlated with memory and learning deficits caused by PCB exposure.^34^ However, these mouse models cannot be used to study the developmental neurotoxicity of PCB 95 because CYP2 and not CYP1 enzymes metabolize PCB congeners with multiple *ortho* chlorine substituents.

Several Cyp2-null mice or CYP2-humanized mice have been reported,^35−38^ and metabolism studies with liver microsomes from *Cyp2f2* and *Cyp2a(4/5)bgs*-null mice confirmed a role of these CYP2 enzymes in the metabolism of PCB 95.^40^ Here we assess the disposition of PCB 95 and its hydroxylated metabolites in male and female *Cyp2abfgs*-null and CYP2A6-transgenic mice in vivo. Furthermore, because the extent of the atropisomeric enrichment is a sensitive and reproducible indicator of differences in the toxicokinetics of chiral PCBs,^41^ we also characterized genotype and sex-dependent differences in the enantiomer fraction (EF) values of the parent PCB. Our results demonstrate that these transgenic mouse models are potential models to study the developmental neurotoxicity of PCB 95, and EF values are a straightforward approach to assess genotype differences in the disposition of PCB 95.

## Materials and Methods

### Chemicals

**Figure 1:**
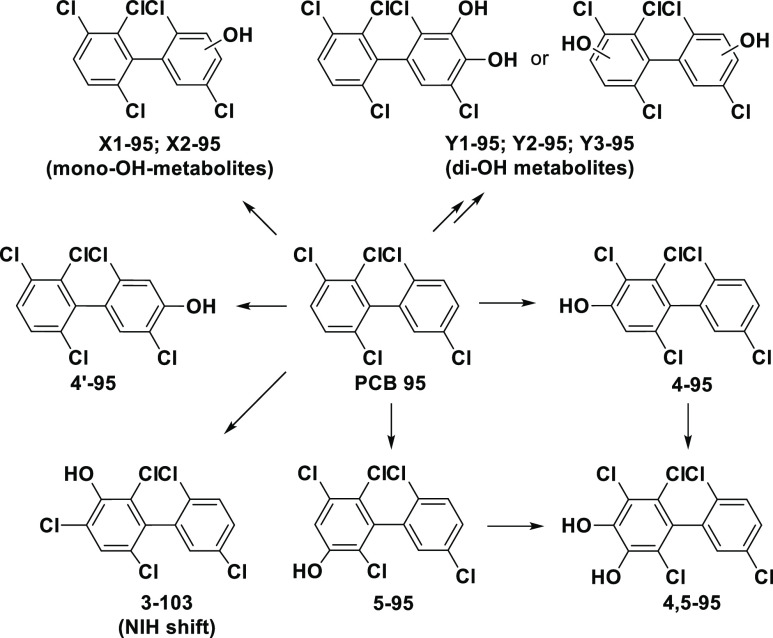
Simplified metabolism scheme of PCB 95. The scheme shows the chemical structures of likely OH-PCB metabolites and the corresponding abbreviations. A summary of all possible hydroxylated PCB 95 metabolites has been reported previously.^69^ X1-95 and X2-95 are unknown mono-hydroxylated metabolites; Y1-95, Y2-95, and Y3-95 are unknown di-hydroxylated metabolites. For unique chemical identifiers, see Table S1.

PCB 95 was synthesized by the Suzuki coupling of 1,2,4-trichloro-3-iodobenzene and 2,5-dichlorobenzeneboronic acid.^42^ Additional information regarding analytical standards and other chemicals, including a list of unique chemical identifiers of all test compounds (Table S1), is provided in the Supporting Information. The abbreviations of the OH-PCB 95 metabolites are a simplification of the PCB metabolite nomenclature proposed by Maervoet and co-workers^43^ and are defined in Figure 1.

### Generation and Maintenance of CYP2A6-Humanized Mouse Models

All animal procedures were approved by the IACUC of the University of Arizona. *Cyp2abfgs*-null (*Cyp2abfgs*^–/–^)^35^ and CYP2A6-transgenic (CYP2A6^+^) mice,^36^ both on a C57BL/6 genetic background, were obtained from breeding colonies maintained at the University of Arizona. To produce the CYP2A6-humanized mouse, *Cyp2abfgs*^–/–^ mice and CYP2A6^+^ mice were intercrossed, yielding CYP2A6^+^/*Cyp2abfgs*^+/–^ pups, which were further intercrossed with *Cyp2abfgs*^–/–^ mice, to generate *Cyp2abfgs*^–/–^ (KO) mice and CYP2A6^+^/*Cyp2abfgs*^–/–^ littermates (CYP2A6-humanized, nicknamed “knock-in” or KI mice). Genotype analysis for the human CYP2A6 transgene and the mouse *Cyp2abfgs* genes were performed as described previously.^35,36^ Mice were provided food and water ad libitum throughout the study and housed in a 12/12 h light/dark cycle in an airflow-, temperature-, and light-controlled environment.

### Immunoblot Analysis of Hepatic CYP2A6 Transgene Expression

The expression of CYP2A6 in KI mice was verified by immunoblot analysis, with use of KO mice as negative controls (Figure S1). Briefly, microsomal proteins were prepared from liver tissues of 2 month-old male and female mice, as described previously.^44^ A mouse anti-CYP2A6 monoclonal antibody (OTI1D2; Invitrogen, Carlsbad, CA) was used to detect CYP2A6. Recombinant human CYP2A6 (Euprotein, North Brunswick, NJ) was used as the standard for CYP2A6 protein detection. For immunoblot analysis, NuPAGE Bis–Tris mini-gels (4–12%) (Invitrogen) were used. Calnexin, a marker protein for the endoplasmic reticulum, was detected using a rabbit anti-calnexin monoclonal antibody (clone 10N19; Sigma-Aldrich, St. Louis, MO) and quantified as a loading control. The secondary antibody was peroxidase-labeled rabbit anti-mouse IgG or goat anti-rabbit IgG and was detected with a SuperSignal West Pico PLUS chemiluminescent substrate (Thermo Scientific, Carlsbad, CA). The intensity of the target band was determined using a Bio-Rad ChemiDoc XRS + imaging densitometer with Image Lab Software (Bio-Rad, Hercules, CA).

### Animal Exposures

Adult male and female wild-type (WT) C57BL/6 mice, KO, and KI mice (4 months old) were exposed to racemic PCB 95. Mice were randomly assigned to exposure groups and received a single oral dose of PCB 95 (1.0 mg/kg) in stripped corn oil (10 mL/kg; lot# A0395699; cat# 801-03-7; Fisher Scientific) via gavage (5–7 mice per group). Control animals received corn oil alone (1–3 mice per group) to assess the potential background contamination with PCB 95. Animals were euthanized 24 h after PCB 95 exposure. Blood was collected from the heart and placed in pre-weighed glass vials with 80 μL of ethylenediaminetetraacetic acid solution (EDTA, 7.5%, w/w). The amount of blood in each vial was determined by weight prior to sample extraction. Adipose, brain, and liver tissues were excised from each animal and individually wrapped in aluminum foil. Samples were stored at −80 °C in separate plastic bags and shipped on dry ice to the University of Iowa for analysis. Body weights of the animals measured before and after PCB dosing, as well as terminal organ weights, are summarized in Table S2.

### Extraction of PCB 95 and Its Metabolites from Tissues

PCB 95 and its hydroxylated metabolites were extracted from adipose (0.06 ± 0.01 g), brain (0.19 ± 0.03 g) and liver tissues (0.49 ± 0.07 g) by pressurized liquid extraction (PLE) with a Dionex ASE200 system (Dionex, Sunnyvale, CA, USA).^45^ Tissues were homogenized with 2 g of pre-cleaned diatomaceous earth (Thermo Fisher Scientific, Pittsburgh, PA, USA) and placed in extraction cells loaded with 10 g of pre-extracted Florisil (60–100 mesh, Fisher Scientific, Pittsburgh, PA, USA). Tissue samples were then spiked with recovery standards [50 ng of PCB 117 in 50 μL of isooctane and 50 ng of 4′-159 (2′,3,3′,5,5′,6′-hexachlorobiphenyl-4-ol) in 50 μL of methanol]. For ongoing precision recovery (OPR) samples; recovery standard, internal standard, PCB 95, and its metabolites (50 ng each) were spiked to the method blank (DE only) and tissues from control animals. Cells were extracted with hexane–dichloromethane–methanol (48:43:9, v/v/v) at 100 °C and 1500 psi (10 MPa), with preheat equilibration for 6 min, 60% of cell flush volume, and 1 static cycle of 5 min. The extracts were concentrated to approximately 1 mL with a TurboVap II (Biotage, LLC, NC, USA) and transferred to new glass tubes with 1 mL of hexane. The solvent was evaporated to near dryness under a gentle stream of nitrogen, and extracts were redissolved in 1 mL of hexane.

### Extraction of PCB 95 and Its Metabolites from Blood

PCB 95 and its hydroxylated metabolites were extracted from whole blood (0.45 ± 0.13 g) using a published liquid–liquid extraction procedure.^29^ Briefly, blood samples were thawed, and 3 mL of aqueous 1% KCl solution was added to each sample. Recovery standards (50 ng of PCB 117 in 50 μL of isooctane and 50 ng of 4′-159 in 50 μL of methanol) were spiked to all samples, including OPR samples, before extraction. Subsequently, 1 mL of 6 M HCl, 5 mL of 2-propanol, and 5 mL of 1:1 hexane–MTBE mixture (v/v) were added to the blood samples. The samples were inverted for 5 min and centrifuged at 1690*g* for 5 min to facilitate the phase separation. Next, the organic phases were transferred to new glass tubes, and the aqueous phases were re-extracted with 3 mL of hexane. Next, three mL of aqueous 1% KCl were added to the combined organic extracts, and samples were inverted and centrifuged as described above. The organic phase was transferred to a new tube, and the aqueous phase was re-extracted with 3 mL of hexane. The combined organic extracts were evaporated to near dryness using a Savant SpeedVac SPD210 (Thermo Scientific, Waltham, MA, US), and each sample was reconstituted with 0.5 mL of hexane.

### Derivatization of OH-PCBs with Diazomethane

Five drops of methanol and 0.5 mL of diazomethane (CH_2_N_2_; about 0.1 mmol) in diethyl ether were added to the tissue or blood extracts to derivatize the OH-PCBs.^45^ Diazomethane rapidly converts the phenolic hydroxyl groups of OH-PCBs to methoxy groups (i.e., −OH → −OCH_3_). All samples were stored at 4 °C for approximately 16 h. Excess diazomethane evaporated under a gentle stream of nitrogen in a fume hood, followed by adding 0.5 mL of hexane. The samples were loaded onto glass SPE cartridges containing 0.2 g of activated silica gel (bottom) and 2 g of acidified silica gel (silica gel/H_2_SO_4_, 2:1, w/w; top). The analytes were eluted with 14 mL of dichloromethane, the eluent was concentrated with a SpeedVac to near dryness, and the solvent was exchanged for hexane. The extracts (approximately 4 mL in hexane) were treated with 4 mL of conc. sulfuric acid for further lipid removal. The organic phase was concentrated to almost dryness with a SpeedVac and spiked with 50 ng of the internal standard (PCB 204 in isooctane) for gas chromatographic quantification of PCB 95 and its hydroxylated metabolites (as methylated derivatives).

### Gas Chromatographic Determinations

PCB 95 and metabolite determinations were conducted on an Agilent 7890B gas chromatograph equipped with an Agilent 7000D Triple Quad and an Agilent 7693 autosampler in the multiple reaction monitoring (MRM) setting. Gas chromatographic separations were performed with an SPB-Octyl capillary column (30 m length, 25 mm inner diameter, 0.25 μm film thickness; Supelco, Bellefonte, PA, USA). Samples were injected in the solvent vent injection mode with a helium (carrier gas) flow of 0.8 mL/min. Nitrogen was used as the collision gas. The following temperature program was used for the separation of PCB 95 and its metabolites: initial temperature of 45 °C, hold for 2 min, 100 °C/min to 75 °C, hold for 5 min, 15 °C/min to 150 °C, hold for 1 min, 2.5 °C/min to 280 °C, and final hold for 5 min. The transfer line temperature was 280 °C. The average relative response factor for the available OH-PCB metabolite standards (as methylated derivatives) was used to estimate the levels of the unknown PCB 95 metabolites.

Atropisomeric analyses of PCB 95 were performed with all samples using an Agilent 7890A gas chromatograph with a ^63^Ni-μECD detector and a Chiralsil-Dex CB (25 m length, 250 μm inner diameter, 0.25 μm film thickness; Agilent, Santa Clara, CA, USA) following a published method with modifications.^48^ The following temperature program was used for the atropisomeric analysis: 50 °C, hold for 1 min, 10 °C/min to 150 °C, hold for 65 min, 15 °C/min to 200 °C, and hold for 15 min. The injector and detector temperature was 250 °C, and the helium flow was 3.0 mL/min. The EF values of PCB 95 were calculated as EF = *A*_E1_/(*A*_E1_ + *A*_E2_) where *A*_E1_ and *A*_E2_ are the peak area of the first (E1) and the second eluting (E2) atropisomers, respectively. E1- and E2-PCB 95 correspond to a*R*- and a*S*-PCB 95, respectively.^16,25^

### Quality Assurance and Quality Control

Method blanks, blank tissue samples, and an ongoing precision and recovery standard were extracted in parallel with each sample batch. The method detection limits (MDLs) were determined based on method blanks (Table S3). Limits of quantification (LOQ) in different matrices were established based on the corresponding tissue blanks (Table S3). Surrogate standards, PCB 117 and 4′-159, were added to all samples to assess the precision and reproducibility of the analytical methods (Table S4). In addition, an ongoing precision and surrogate standard was in parallel extracted from both method and tissue blanks with each sample batch (Table S5). The resolution of atropisomers of PCB 95 on the Chiralsil Dex CB column was 1.27, calculated with the formula Rs = [*t*_E2_ – *t*_E1_)/(0.5 × (*W*_E1_ + *W*_E2_)], where *t*_E1_ and *t*_E2_ are the retention times of peaks 1 and 2, and *W*_E1_ and *W*_E2_ indicate the width of the peak E1 and E2. The EF value of the racemic standard of PCB 95 was 0.498 ± 0.004 (*n* = 7).

### Data Analysis

Levels of PCB 95 and its metabolites, expressed as ng per gram wet weight (ng/g), and EF values are reported as mean ± standard deviation (Tables S6 and S7). The statistical analyses were performed with two-way ANOVA with the Bonferroni model in GraphPad Prism 9.4.1 (Tables S8 and S9). In addition, OH-PCB metabolite profiles were compared using the similarity coefficient, cos θ (Tables S10 and S11).^49^ The cos θ ranges from 0 to 1, where a value of 0 indicates completely different profiles and a value of 1 indicates identical profiles. The original data underlying this study are openly available through the Iowa Research Online repository at https://doi.org/10.25820/data.006613.

## Results and Discussion

### Comparison of PCB 95 Tissue Levels

**Figure 2:**
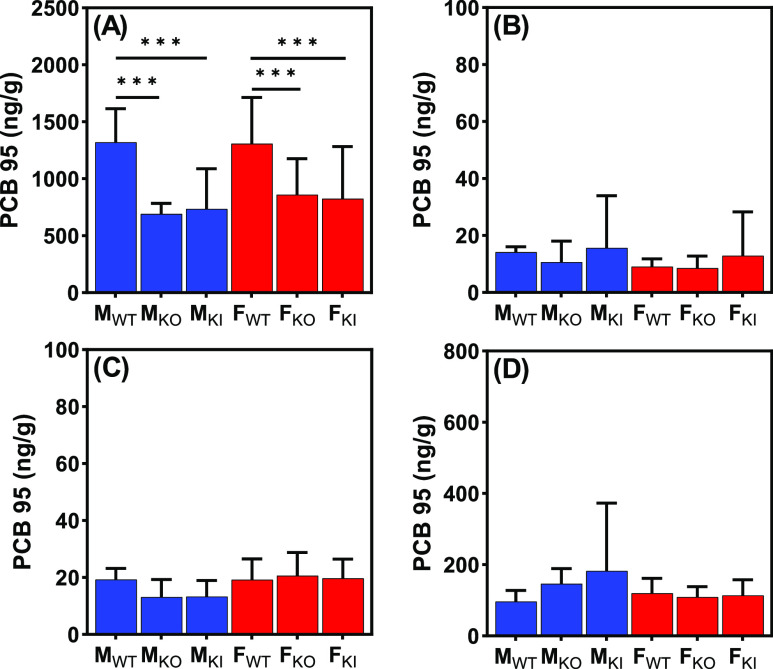
PCB 95 tissue levels. A comparison of the levels of PCB 95 (ng/g tissue) in (A) adipose, (B) blood, (C) brain, and (D) liver from male and female wildtype, *Cyp2abfgs*-null, and CYP2A6-humanized mice reveals genotype-dependent differences in the disposition of PCB 95 in the adipose tissue only. Data are averages ± SD and presented on a logarithmic scale; for the actual PCB 95 levels, see Table S6. ***: *p* < 0.0001 (for other *p*-values, see Table S8). Statistical analyses were performed by using the two-way ANOVA analysis tool with the Bonferroni correction for multiple comparisons in GraphPad Prism 9.4.1. M_WT_, male wildtype mice; M_KO_, male *Cyp2abfgs*-null mice; M_KI_, male CYP2A6-humanized mice; F_WT_, female wildtype mice; F_KO_, female *Cyp2abfgs*-null mice; F_KI_, female CYP2A6-humanized mice.

PCB 95 levels followed the rank order adipose > liver > blood–brain in all six exposure groups (Figure 2), consistent with the fat content of these tissues. The mean adipose levels were 4- to 14-times higher than the liver and 42- to 68-times higher than the blood levels. Other PCB 95 disposition studies have reported a similar rank order in PCB 95 tissue levels in mice^48,50^ or rats.^22^ Because PCBs are lipophilic compounds, their partitioning into tissues is expected to correlate with the fat content of the tissue, a fact that can be used to approximate the partitioning of PCBs from the blood into target tissues based on the tissue composition.^30^ In this study, the fold difference between adipose and liver PCB 95 levels is consistent with an approximately 8-fold difference in the extractable lipid content between both tissues in the mouse (8.8 and 72%, respectively^29^). In contrast, the extractable lipid content of the brain and liver are comparable (8.8 vs 9.3%, respectively), but the PCB 95 levels are lower in the brain than in the liver. These differences likely reflect differences in the lipid composition of the brain compared to other tissues or a dysfunction of the blood brain barrier.

### PCB 95 Levels in the Adipose

Levels of PCB in the adipose tissue ranged from 690 ng/g for M_KO_ to 1300 ng/g for M_WT_ and F_WT_ mice (Figure 2 and Table S6). These levels are lower than those observed in other animal studies using sub-acute or sub-chronic PCB exposure paradigms. For example, PCB 95 levels in the adipose tissue of female mice after sub-chronic oral exposure to different doses of PCB 95 ranged from 1800 ng/g (0.1 mg/kg bw/d) to 47,000 ng/g (6.0 mg/kg bw/d).^48^ Somewhat lower PCB 95 levels were reported for the adipose tissue of dams exposed to PCB 95 during gestation and lactation, ranging from 170 ng/g at the low dose (0.1 mg/kg bw/d) to 11,600 ng/g at the high dose (6.0 mg/kg bw/d), reflecting growth dilution of the total PCB 95 dose during pregnancy.^50^ In male Wistar rats, PCB 95 levels were 9300 ng/g in the adipose tissue following sub-acute, oral PCB 95 exposure.^22^ Overall, the higher PCB 95 adipose levels following repeated doses are not surprising because adipose tissue is a storage site for PCBs because of its high-fat content. PCB 95 levels in human adipose tissue have been rarely reported, possibly because PCB 95 is more rapidly metabolized by human cytochrome P450 enzymes, such as CYP2A6 and CYP2B6,^26^ than more persistent PCB congeners. For example, PCB 95 was below the limit of detection in human adipose tissue collected from 2008 to 2009 during abdominal operations in Southeast China.^51^ Only 20 PCB congeners were frequently detected in these samples. PCB 95 was also below the detection limit in breast adipose tissue collected in 2001 from Japanese women.^52^

### PCB 95 Levels in the Blood

The PCB 95 levels in blood ranged from 9 ng/g in F_KO_ mice to 16 ng/g in M_KI_ mice 24 h after PCB 95 exposure (Figure 2 and Table S6). Comparable PCB 95 levels of 36 ng/g were observed in postnatal day 21 pups exposed to PCB 95 throughout gestation and lactation via the maternal diet.^24^ The mean PCB 95 blood level in the corresponding dams was 16 ng/g. In comparison, PCB 95 levels in the blood of female mice after subchronic oral exposure to different doses of PCB 95 ranged from 90 ng/g (0.1 mg/kg bw/d) to 1200 ng/g (6.0 mg/kg bw/d).^48^ Lower PCB 95 levels were observed in the blood of dams exposed orally to PCB 95 during gestation and lactation. Levels in these animals ranged from not detected at the low dose (0.1 mg/kg bw/d) to 52 ng/g at the high dose (6.0 mg/kg bw/d).^50^ In male Wistar rats, PCB 95 levels were 9 ng/g in the blood following sub-acute, oral PCB 95 exposure.^22^ Animals in these published studies were typically euthanized approximately 24 to 28 h after the last PCB 95 administration.

Unfortunately, PCB 95 is not frequently analyzed in human biomonitoring studies, partly because it has a low detection frequency.^53,54^ For example, PCB 95 was detected in only one sample at a concentration of 0.1 ng/g wet weight in a study of maternal serum from the high-risk autism spectrum disorder MARBLES cohort.^53^ PCB 95 was also below the detection limit in serum collected in 2001 from Japanese women.^52^ The total PCB levels in the Japanese cohort ranged from 1.6 to 6.9 ng/g serum and are lower compared to the PCB 95 levels observed in animal studies. The relatively low detection frequency of PCB 95 in serum is not surprising because PCB 95 readily partitions from the blood into tissues,^30,55^ and as shown in mice, is cleared relatively quickly^56^ due to its metabolism by cytochrome P450 enzymes.^26^ Only one study of a cohort of highly exposed e-waste workers detected PCB 95 at comparatively high levels, with a detection frequency of 92%, thus enabling the assessment of its enantiomeric enrichment in this cohort.^54^

### PCB 95 Levels in the Brain

PCB 95 levels in the whole brain of mice ranged from 13 to 21 ng/g wet weight across all three genotypes and for both sexes (Figure 2 and Table S6). Information about PCB 95 brain levels following acute exposure of rodents to PCB 95 remains limited. More data about PCB 95 levels in the rodent brain are available from sub-acute and sub-chronic PCB 95 disposition studies. For example, PCB 95 levels in the female mouse brain after 39 days of daily oral administration of different doses of PCB 95 (0.1 to 6.0 mg/kg bw/d) were higher than in the present study and ranged from 37 to 360 ng/g.^24,48^ PCB 95 levels in the brain of mice exposed orally to different PCB 95 doses throughout pregnancy were below the detection limit of 140 ng/g.^50^ The levels in the offspring of pregnant mice exposed developmentally to PCB 95 were 50 and 70 ng/g on postnatal days 7 and 21.^24^ These levels were comparable to the levels of 47 ng/g PCB 95 observed in the dams euthanized on postnatal day 21. In male Wistar rats, PCB 95 levels were 51 ng/g in the cerebellum and 29 ng/g in the cortex following sub-acute, oral PCB 95 exposure.^22^ These earlier studies were part of larger animal experiments investigating the developmental neurotoxicity of PCBs, and similar to the present acute exposure study used PCB doses considered to be environmentally relevant (i.e., 0.1 to 6 mg/kg bw/d) and neurotoxic.^19,57,58^

PCB levels, including tissue wet weights-adjusted PCB 95 levels, have rarely been determined in the human brain. The average PCB 95 levels in postmortem brain tissues from older donors aged 58 to 80 from the United States were 0.13 ng/g (ranging from not detected to 0.14 ng/g, with a detection frequency of 3%).^12^ PCB 95 was not detected in younger donors aged 0 days to 1 year in the same study. Like human blood samples, as discussed above, PCB 95 was also seen with a low detection frequency of 11% in postmortem brain tissues from donors in the United States.^11^ Total PCB levels reported for postmortem human brain samples range from 1.9 to 7.0 ng/g for donors from Poland,^59^ 12 ng/g for one sample from Belgium,^60^ and not detected to 2.8 ng/g for neonates and 0.03 to 3.1 ng/g for adult donors from the United States.^12^ Higher total PCB levels were reported for two older British males (64 and 84 ng/g)^61^ and a Yucheng patient in Taiwan (80 ng/g).^62^ This comparison reveals that the PCB 95 levels observed in the present study are close to the range of the brain levels observed in some human populations.

### PCB 95 Levels in the Liver

PCB 95 liver levels ranged from 96 ng/g in M_WT_ mice to 183 ng/g in M_KI_ mice (Figure 2 and Table S6). Similarly, PCB 95 levels in the liver of dams exposed throughout gestation and lactation were 120 ng/g wet weight.^24^ The levels in the corresponding offspring on postnatal day 21 were 150 ng/g wet weight. In contrast, PCB 95 levels in the liver of female mice after sub-chronic oral exposure to different doses of PCB 95 ranged from 90 ng/g (0.1 mg/kg bw/d) to 1200 ng/g (6.0 mg/kg bw/d).^48^ Levels of PCB 95 in the liver of dams exposed to racemic PCB 95 during gestation and lactation ranged from 12 ng/g at the low dose (0.1 mg/kg bw/d) to 700 ng/g at the high dose (6.0 mg/kg bw/d).^50^ In male Wistar rats, PCB 95 levels were 120 ng/g in the liver following sub-acute, oral PCB 95 exposure.^22^ Unfortunately, limited information about levels of PCB 95 and other congeners in the human liver is available, even though PCB exposure is implicated in human liver disease.^63^ One study from Belgium reports that PCB 95 was below the limit of detection in liver samples from a small human cohort (*N* = 11).^60^

### Effects of Genotypes on PCB 95 Levels

For both male and female mice, PCB 95 adipose levels were significantly higher in WT than in KO or KI mice (Figure 2 and Table S6). The higher PCB 95 levels in the adipose tissue from WT than KO or KI mice appear inconsistent with a *Cyp2abfgs*-dependent disposition of PCB 95 in WT animals. Instead, the knockout of *Cyp2abfgs* and the knock-in of CYP2A6 may affect the composition and/or size of fat depots or other physiological parameters that affect the toxicokinetics of PCB 95 in KO and KI mice. Similarly, we have shown that the liver-specific deletion of cytochrome P450 reductase results in a fatty liver that drastically alters the disposition of PCBs.^29,30,41^ These possibilities need to be explored in additional toxicokinetic studies.

### Atropisomeric Enrichment of PCB 95 in Tissues

**Figure 3:**
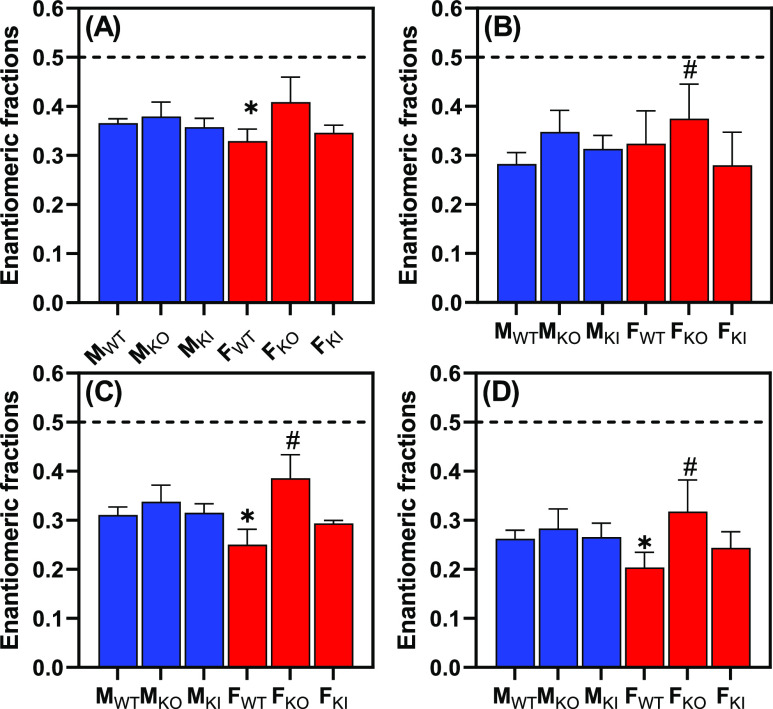
A comparison of the EFs of PCB 95 (ng/g tissue) in (A) adipose, (B) blood, (C) brain, and (D) liver from male and female wildtype, *Cyp2abfgs*-null, and CYP2A6-humanized mice reveals sex and genotype-dependent differences in the atropselective disposition of PCB 95, with the PCB 95 atropisomer eluting second on the chiral gas chromatography column being enriched in all tissues. The EF values of PCB 95 were calculated as EF = *A*_E1_/(*A*_E1_ + *A*_E2_) where *A*_E1_ and *A*_E2_ are the peak area of the first (E1-PCB 95) and the second eluting (E2-PCB 95) atropisomer of PCB 95, respectively. E1- and E2-PCB 95 correspond to a*R*- and a*S*-PCB 95, respectively.^16,25^ The data are reported as average ± SD; for the EF values, see Table S7. The dotted line indicates the EF value (0.5) of racemic PCB 95. Statistical analyses were performed by using the two-way ANOVA analysis tool with the Bonferroni correction for multiple comparisons in GraphPad Prism 9.4.1. * Significantly different from KO mice, ^#^ significantly different from KI mice (for the *p*-values, see Table S9). M_WT_, male wildtype mice; M_KO_, male *Cyp2abfgs*-null mice; M_KI_, male CYP2A6-humanized mice; F_WT_, female wildtype mice; F_KO_, female *Cyp2abfgs*-null mice; F_KI_, female CYP2A6-humanized mice.

The atropisomeric enrichment of chiral PCBs, such as PCB 95, is a powerful approach to studying the disposition of PCBs in vitro and in vivo.^13,64,65^ For example, the extent of the atropisomeric enrichment of PCB 95 will depend on the degree to which PCB 95 is metabolized to hydroxylated and other metabolites. Moreover, EF values are a straightforward indicator of subtle differences in the toxicokinetics of the different PCB atropisomers.^41^ In the present study, we observed an enrichment of the PCB 95 congener eluting second on the chiral gas chromatography column in all tissues investigated (Figures 3 and S2–S3). This PCB 95 atropisomer corresponds to a*S*-PCB 95 (or (+)-PCB 95).^16,25^ Similarly, other PCB 95 disposition studies report enrichment of a*S*-PCB 95 in mouse tissues.^21,48,50^ In contrast, in vitro metabolism of PCB 95 by human cytochrome P450 enzymes, including CYP2A6, results in an enrichment of a*R*-PCB 95 (or (−)-PCB 95).^21,25^

The EF values in the tissues from F_KO_ mice were significantly larger than those in the F_WT_ mice, with a statistically significant difference for adipose, brain, and liver tissues (Figure 3 and Table S7). These differences are consistent with a more extensive metabolism of PCB 95, evidenced by a less pronounced atropisomeric enrichment (i.e., EF value closer to the EF of racemic PCB 95), in F_WT_ than F_KO_ mice, consistent with an impaired PCB 95 metabolism in F_KO_ mice due to the deletion of *Cyp2abfgs*. Similarly, the EF values in all tissues were smaller in F_KI_ than those in F_KO_ mice. This difference in the EF values was statistically significant for blood, brain, and liver tissues, an observation consistent with a more pronounced metabolism of PCB 95 in F_KI_ than F_KO_ mice. However, because CYP2A6 preferentially metabolizes a*S*-PCB 95, the direction of the atropisomeric enrichment observed in KI mice does not suggest a significant contribution of CYP2A6 to the metabolism of PCB 95. Instead, mouse cytochrome P450 enzymes other than the disrupted Cyp2 enzymes may contribute to the atropisomeric enrichment of PCB 95 in KI mice. As we have proposed for in vitro metabolism studies with liver microsomes from other Cyp2-null mice, an in-depth proteomic characterization of mouse cytochrome P450 profiles is needed to fully characterize PCB metabolism in mice.^40^

Interestingly, no genotype-dependent differences in the EF values of PCB 95 were observed in male mice. This observation appears to contradict our observation of significant differences in PCB 95 levels in male mice (Figure 2). However, the significantly different PCB levels in male mice may mask genotype-specific differences to the extent of the atropselective metabolism of PCB. As discussed above, detailed toxicokinetic studies are needed to determine how the altered cytochrome P450 enzyme profiles in KO and KI mice alter the metabolism of the PCB 95 atropisomers.

### Identification of Hydroxylated PCB 95 Metabolites

**Figure 4:**
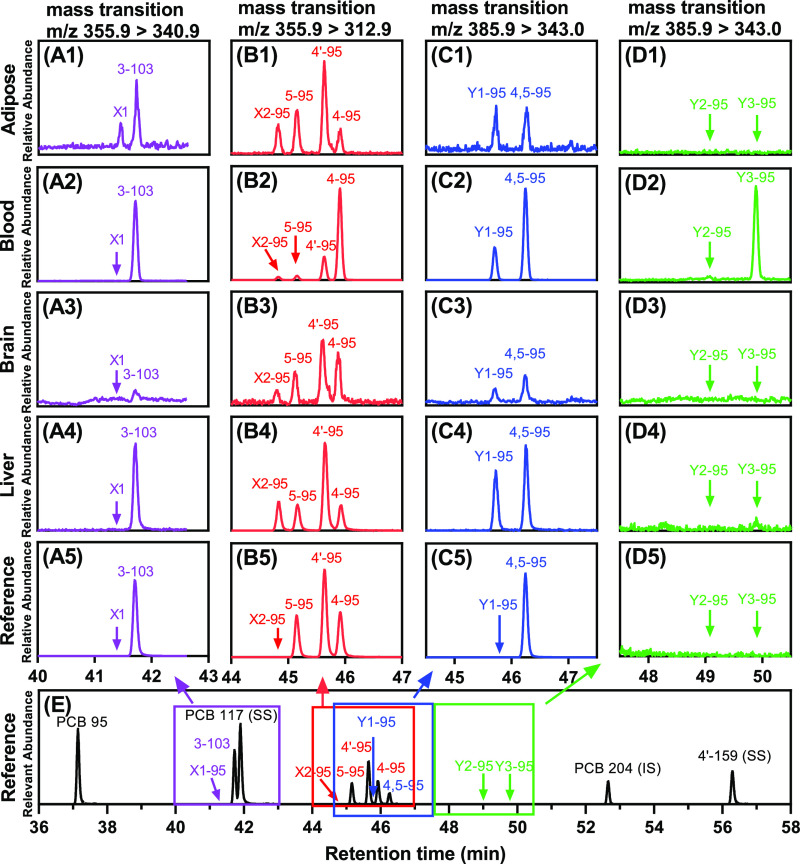
Detection of PCB 95 metabolites. Representative chromatograms show the presence of six mono- and four di-hydroxylated metabolites, analyzed as the corresponding methoxylated derivatives, in (panel A1–D1) adipose, (panel A2–D2) brain, (panel A3–D3) liver, and (panel A4–D4) blood of a PCB 95 exposed female knock-in (F_KI_) mouse. (A5–D5) The chromatograms of authentic standards in the reference sample are shown for comparison. (E) The total ion chromatogram (TIC) of the reference sample which was with surrogate standards (SSs, PCB 117 and 4′-159), internal standard (IS, PCB 204), and PCB 95, 5-95, 4′-95, 4-95, and 4,5-95. The extracted chromatograms show mono-hydroxylated PCB 95 metabolites with mass transition of *m*/*z* 355.9 → 340.9 (panels A1–A5), *m*/*z* 355.9 → 312.9 (panels B1–B5) and di-hydroxylated PCB 95 metabolites with mass transition of *m*/*z* 385.9 → 343.0 (panels C1–C5 and D1-D5). Analyses were performed by GC–MS/MS as described under Materials and Methods. X1-95 and X2-95 indicate the unknown monohydroxylated PCB 95 metabolites, and Y1, Y2, and Y3 indicate the unknown dihydroxylated PCB 95 metabolites.

Because PCB 95 is metabolized to hydroxylated metabolites, we also determined OH-PCB levels in adipose, blood, brain, and liver tissues. Six mono-hydroxylated and four di-hydroxylated metabolites of PCB 95 were detected in blood and tissues across all six mouse groups (Figures 1 and 4). Three metabolites, 4-95, 4′-95, and 5-95, were identified using authentic standards. These metabolites were also detected in earlier PCB 95 disposition studies in mice.^24,48,50^ A 1,2-shift metabolite, 3-103, was a minor metabolite detected in a few blood and tissue samples, consistent with previous disposition studies.^24,48,50^ Two unknown mono-hydroxylated metabolites, X1-95 and X2-95, were also detected and tentatively identified as mono-hydroxylated PCB 95 metabolites based on their mass transition in the MRM mode. We tentatively identified these metabolites based on the retention time relatively to the available analytical standards (Figure 4). Briefly, mono-hydroxylated metabolites of PCB 95 and structurally related PCB congeners, analyzed as the corresponding methoxylated PCBs, typically have similar relative retention times. In contrast, the corresponding 1,2-shift products have a much shorter retention time (e.g., Figure 4B).^26,41,66^ Therefore, X1-95 likely corresponds to a 1,2-shift metabolite in the 2,5-dichlorophenyl ring. Because the retention time of methoxylated PCBs follows the order meta < para hydroxylated PCB metabolites,^26,41,66,67^ X2-95 may correspond to 3′-95, the only meta/para hydroxylate metabolite for which no analytical standard was available.

One di-hydroxylated metabolite, 4,5-95, was identified using an authentic standard. Several other studies also report the formation of 4,5-95 in mice.^24,48,50^ Three unknown dihydroxylated metabolites, Y1- through Y3-95, were also detected. The retention time of Y1-95 suggests that this metabolite is likely a catechol metabolite, such as 3′,4′-95. The comparatively long retention times of Y2-95 and Y3-95 indicate that these metabolites are hydroxylated in different rings of the biphenyl moiety. Analogous dihydroxylated metabolites have been reported in the feces of mice, rats, and quails exposed to PCB 95.^68^ Based on the metabolites detected, we propose the simplified PCB 95 metabolism scheme shown in Figure 1. This metabolism scheme is consistent with the earlier PCB disposition studies in mice^24,48,50^ and other animal models.^21,68,69^ Additional PCB 95 metabolites were likely present but could not be detected due to the targeted GC–MS/MS analysis approach or because the metabolites (e.g., sulfate and other conjugates^70^) are not amenable to gas chromatographic analysis.

### Comparison of OH-PCB Profiles

Comparisons of the OH-PCB chromatograms (Figure 4) and the OH-PCB profiles using the similarity coefficient cos θ (Tables S10 and S11) revealed differences in the disposition of OH-PCB 95 metabolites across tissues. Moreover, slight differences by genotypes and sex were observed. In particular, sex- and tissue-dependent differences in PCB metabolite tissue profiles have not been reported previously, partly because earlier PCB 95 disposition studies only reported a few hydroxylated metabolites. In the present study, the OH-PCB profiles in the liver and blood of male and female mice were similar across all three genotypes (cos θ ≥ 0.93). However, for both male and female mice, the OH-PCB profiles in the adipose tissue from KO mice differed from those in WT and KI mice, a difference that was more pronounced in female than male mice. Moreover, the brain OH-PCB profiles in WT mice differed from those observed in KO and KI mice (cos θ ranging from 0.46 to 0.78). The brain OH-PCB profiles also differed between KO and KI mice (cos θ of 0.78 for male and cos θ of 0.83 for female mice). In addition, the OH-PCB profiles showed sex differences in adipose and brain tissues from all three genotypes (cos θ ranging from 0.56 to 0.90). Finally, OH-PCB profiles differed between adipose, blood, and liver tissues from animals with the same genotype. These differences were more pronounced in female than male mice (cos θ ranging from 0.43 to 0.95 for male and cos θ from 0.29 to 0.77 for female mice). The differences in the OH-PCB profiles in different tissues likely reflect genotype-dependent differences in the toxicokinetics of individual OH-PCB metabolites. This result suggests a need to further explore PCB metabolite profiles in target tissues, for example, using nontargeted high-resolution mass spectrometry approaches.^71,72^

### Total OH-PCB Blood and Tissue Levels

The highest total OH-PCB metabolite levels were observed in whole blood (Table S6). Across all genotypes, blood OH-PCB levels ranged from 20 ng/g in M_KI_ to 45 ng/g in M_WT_ mice. Comparable or higher total OH-PCB levels were reported for repeated dose studies investigating the disposition of PCB 95 in rodents exposed orally or via the maternal diet.^22,24,48,50^ Comparing these OH-PCB levels with human biomonitoring studies is challenging because these studies typically use serum or plasma, not whole blood. A study of the Canadian Inuit reported that total OH-PCB in this population varies by orders of magnitude, with whole blood levels as high as 12 ng/g.^73^ However, the mean total OH-PCB levels in this population were lower than in animal studies.

In the other tissues investigated, total OH-PCB levels followed the rank order liver > adipose > brain (Table S6). Similar to this study, higher blood than liver OH-PCB levels have been observed in female or pregnant mice exposed orally to PCB 95.^48,50^ The observation that several OH-PCBs were observed in adipose tissue is intriguing because earlier studies with PCB did not detect OH-PCBs in the adipose tissue.^48,50^ Depending on the genotype and sex, total OH-PCB levels in adipose tissue were 2.6 to 10-fold lower than those in whole blood (Table S6). OH-PCBs were also observed in adipose tissue of women undergoing breast cancer surgery in Japan in 2001; total OH-PCBs in adipose were 4–5 times lower than those in serum in this human biomonitoring study.^52^

OH-PCBs were detected with low detection frequencies and at low levels in the brain (Table S6), an observation consistent with other PCB 95 disposition studies in rodents.^48,50^ One exception is a disposition study in dams and pups exposed to PCB 95 via the maternal diet, which reported that PCB 95 metabolites are present in the brain of these mice, with total OH-PCB levels ranging from 2.4 ng/g in dams to 10 ng/g in postnatal day pups.^24^ These different observations are likely due to the different instrumentation used across studies (i.e., GC–MS/MS in this study and gas chromatography with electron capture detection in the earlier studies). It is also possible that the levels of PCB 95 metabolites in the brain are highly transient, as suggested by a disposition study of PCB 11 sulfate metabolites,^74^ i.e., some studies may have analyzed brain tissue at a time point before or after the maximum tissue concentration of the PCB 95 metabolites was achieved.

**Figure 5:**
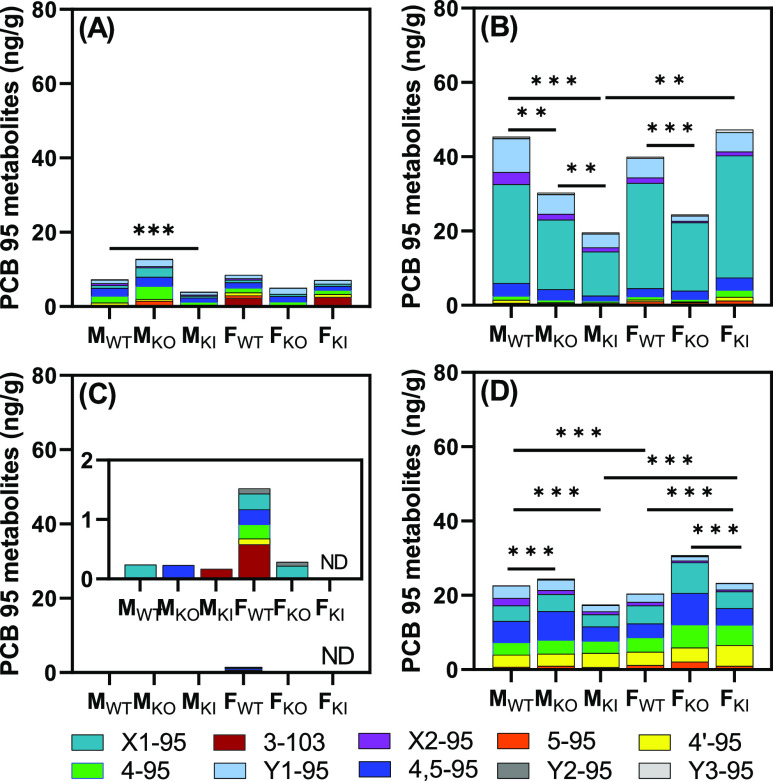
PCB 95 metabolite profiles. A comparison of the profiles and levels of the hydroxylated PCB 95 metabolites (ng/g tissue) in (A) adipose, (B) blood, (C) brain, and (D) liver from male and female WT, KO, and KI mice reveals sex and genotype-dependent differences in the disposition of hydroxylated PCB 95 metabolites. Statistical analyses were performed by using the two-way ANOVA analysis tool with the Bonferroni correction for multiple comparisons in GraphPad Prism 9.4.1; ***: *p* < 0.0001, **: *p* < 0.001 (Table S8). M_WT_, male wildtype mice; M_KO_, male *Cyp2abfgs*-null mice; M_KI_, male CYP2A6-humanized mice; F_WT_, female wildtype mice; F_KO_, female *Cyp2abfgs*-null mice; F_KI_, female CYP2A6-humanized mice; ND, not detected.

Genotype- and sex-dependent differences in total OH-PCB levels were observed in the blood and liver (Figure 5). In blood, total OH-PCB metabolite levels were lower in male and female KO than those in WT mice. The opposite trend was observed in the liver, with higher total OH-PCB metabolite levels in KO than in WT mice. This difference was only significant in the liver of male mice. Moreover, in male mice, total OH-PCB metabolite levels were significantly lower in M_KI_ than those in M_WT_ mice in the blood and liver. The opposite trend was observed in the blood and liver of female mice, with total OH-PCB metabolite levels being higher in F_KI_ than those in F_WT_ mice. The only sex difference in total OH-PCB metabolite levels was observed in the blood and liver of KI mice, with higher levels present in F_KI_ than in M_KI_ mice. In an earlier study, no statistically significant sex differences in OH-PCB levels were observed in mouse pups exposed to PCB 95 via the maternal diet.^24^ It is possible that the repeated dosing paradigm in the published study masked sex differences that are apparent with the acute dosing paradigm in this study. In contrast, sex differences in OH-PCB levels have been reported in a human biomonitoring study, where PCB metabolite levels were higher in men than those in women.^75^

### Detection Frequencies and Levels of Individual OH-PCB Congeners in Adipose Tissue

Two PCB congeners, 5-95 and 4′-95, were consistently detected in the adipose tissue across all six exposure groups (Table S6). The unknown metabolite, X1-95, was detected in all male and female WT and KO mice but had a lower detection frequency in KI mice. Other metabolites were observed with detection frequencies ranging from not detected to approximately 85%, likely because of the low levels of OH-PCB metabolites in the adipose tissue. Overall, 5-95 and, when detected, 4,5-95 had the highest levels in the adipose tissue. Other rodent studies did not report the presence of OH-PCBs in adipose tissue,^48,50^ possibly because of the different dosing paradigms (i.e., acute vs repeated dose), sample collection time points, or analytical methods employed. In contrast, OH-PCBs of higher chlorinated PCBs have been detected in human adipose tissue.^52,76,77^ Because OH-PCB metabolites structurally related to 4′-95, such as 4-52 (2,2′,5,5′-tetrachlorobiphenyl-4-ol), can have profound effects on preadipocytes in vitro,^78^ further studies are needed to assess the presence of OH-PCBs in various fat depots in both laboratory animals and humans.

### Detection Frequencies and Levels of Individual OH-PCB Congeners in Whole Blood

All OH-PCB metabolites were detected in all blood samples across all exposure groups, except for 3-103 and Y2-95. Interestingly, Y1-95 appeared to be a major PCB 95 metabolite observed in the blood from all exposure groups, which needs to be confirmed using an authentic standard. The levels of metabolites with available standards followed the rank order 4,5-95 > 4′-95 > 4-95 > 5-95 > 3-103 (Table S6). Different rank orders of these OH-PCB 95 metabolites were reported in mice and rats exposed repeatedly to PCB 95.^22,24,48,50^ These studies underscore that blood PCB metabolite profiles depend on numerous factors, including the dosing paradigm and species. Statistical analyses revealed no significant sex and genotype differences in individual OH-PCB levels (Table S8). Thus, the genotype-dependent differences in total OH-PCB blood levels shown in Figure 5 may reflect general, across-the-board effects of the deletion of *Cyp2abfgs* genes and the knock in of human CYP2A6 on the oxidation of PCB 95 to OH-PCB.

### Detection Frequencies and Levels of Individual OH-PCB Congeners in the Liver

Most OH-PCB metabolites were detected in the liver across all exposure groups (Table S6). Y2-95 and Y3-95 were observed sporadically across exposure groups, and X1-95 was essentially not detected in the liver. The levels of metabolites with available standards were quite similar and ranged from 0.4 ng/g tissue for 4-95 in F_KI_ mice to 8.5 ng/g tissue for 4′-95 in F_KO_ mice. As a result, no consistent rank order was observed across the six exposure groups. However, the major metabolites were 4′-95, 5-95, and 4,5-95. As in blood, Y1-95 was also a major metabolite in the liver. In addition, 4-95 and 3-103 were minor metabolites in the liver. These findings are consistent with earlier studies of the disposition of PCB 95 metabolites in rodents.^22,24,48,50^ Interestingly, genotype- and sex-dependent differences were observed for 4′-95 and 5-95 (Table S7). For example, 4-95 levels were higher in F_WT_ than those in F_KI_ mice. Moreover, 4′-95 and 4-95 levels were higher in male than those in female WT mice. Significant differences in metabolite levels were also observed for KI mice, with 4′-95 levels higher in F_KI_ than those in M_KI_ mice and 4-95 levels lower in F_KI_ than those in M_KI_ mice.

### Potential Application of KO and KI Mice for Developmental Neurotoxicity Studies

The present study provides novel, fundamental insights into the atropselective disposition of PCB 95 and its hydroxylated metabolites in mice, and the usefulness and limitations of KO or KI mice for developmental neurotoxicity studies. Tissue levels of PCB 95 and its metabolites, and EF values of PCB 95, were similar to or lower than those observed in other animal studies using environmentally relevant sub-acute or sub-chronic PCB 95 exposure paradigms. PCB 95 levels in this and other animal studies tended to be higher than the limited human PCB 95 tissue levels reported in the literature; however, brain PCB levels in rodents can be similar to total PCB levels in some human populations. The metabolites identified in the tissues investigated were consistent with published rodent disposition studies. Like parent PCB 95, total OH-PCB levels in human tissues are typically lower than those in rodent studies. PCB 95 metabolites have not been reported in human samples to date. These species comparisons must be interpreted cautiously because, unlike animal studies, exposure levels and times are generally unknown in humans.

Our analysis also revealed several genotype-dependent differences in PCB 95 and its metabolite levels. Briefly, the genotype only affected PCB 95 levels in adipose tissue, with higher PCB 95 adipose levels in WT than in KO and KI mice. In addition, genotype-dependent differences were observed for liver levels of two metabolites, 4′-95 and 4-95. These results suggest that the deletion of *Cyp2abfgs* and, subsequently, the knock-in of the human CYP2A6 transgene alters PCB 95 toxicokinetics in mice. Several changes in the PCB 95 disposition across the three genotypes appear inconsistent with the role of CYP2ABFGS or human CYP2A6 enzymes in the disposition of PCB 95 in mice. However, it is important to emphasize that the CYP2 enzymes disrupted in KO mice and the human CYP2A6 in KI mice are not the only enzymes responsible for PCB 95 metabolism in the mouse liver. Moreover, the metabolite levels were affected not only by the levels or availability of the CYP enzymes but also by the bioavailability of PCB 95 to the liver, which is affected by PCB 95 levels stored in the fat and other organs (which was variable among the groups) and the rate of redistribution from these stores to the liver for metabolism.

Finally, the atropisomeric enrichment of PCB 95 revealed two important findings. First, the direction of the atropisomeric enrichment in KI mice is the same as in WT and KO mice. Thus, the direction of the atropisomeric enrichment in KI mice differs from that observed in metabolism studies with recombinant CYP2A6 or human liver microsomes. In addition to human CYP2A6, mouse cytochrome P450 enzymes other than the disrupted CYP2 enzymes likely contribute to the atropisomeric enrichment of PCB 95 in KI mice. Therefore, an in-depth characterization of mouse cytochrome P450 profiles is needed to fully characterize PCB 95 metabolism in mice. Second, the atropselective analysis revealed genotype and sex differences in the disposition of PCB 95. Briefly, KO mice displayed a less pronounced atropisomeric enrichment than female WT and KI mice, consistent with a less pronounced metabolism of PCB 95 in KO. The results from the atropselective analysis demonstrate that EF values are a powerful tool to assess differences in the toxicokinetics of chiral PCBs in mice. Overall, KO mice hold promise to study the role of CYP2 enzymes in PCB 95 developmental neurotoxicity. However, additional work is needed to determine to which extent KI mice are a good model for the metabolism of PCB 95 in humans.

## Supporting Information Available

Unique chemical identifiers of analytical standards, animal body weights and tissue weights, QA/QC information, levels of PCB 95 and its hydroxylated metabolites in tissues, enantiomeric fractions of PCB 95 in tissues, summaries of *p*-values from the statistical analyses, similarity coefficients comparing metabolite profiles across genotypes and tissues, hepatic expression of transgenic CYP2A6, and representative chromatograms showing an enrichment of the second eluting atropisomer of PCB 95 in tissues (PDF)
