# Supplementary material for: Enantiomeric Fractions Reveal Differences in the Atropselective Disposition of 2,2′,3,5′,6-Pentachlorobiphenyl (PCB 95) in Wildtype, Cyp2abfgs-Null, and CYP2A6-Humanized Mice
Source: Chem Res Toxicol. 2023 Jul 19;36(8):1386–97. doi: 10.1021/acs.chemrestox.3c00128 (PMC10445290; doi:10.1021/acs.chemrestox.3c00128)
Supplement: Supplementary file 1 — tx3c00128_si_001.pdf [file tx3c00128_si_001.pdf]

Supporting Information

Enantiomeric Fractions Reveal Differences in the Atropselective Disposition of 2,2',3,5',6-Pentachlorobiphenyl (PCB 95) in Wildtype, *Cyp2abfgs*-null and CYP2A6-Humanized Mice

Xueshu Li,[1] Amanda J Bullert,[1,2] Weiguo Han,[3] Weizhu Yang,[3] Qing-Yu Zhang,[3] Xinxin Ding,[3] Hans-Joachim Lehmler[1,2,*]

[1] Department of Occupational and Environmental Health, College of Public Health, University of Iowa, Iowa City, IA 52242, USA; [2] Interdisciplinary Graduate Program in Neuroscience, University of Iowa, Iowa City, IA 52242, USA; [3] Department of Pharmacology and Toxicology, College of Pharmacy, University of Arizona, Tucson, Arizona 85721, USA.

Corresponding Author:
Dr. Hans-Joachim Lehmler
The University of Iowa
Department of Occupational and Environmental Health
University of Iowa Research Park, #221 IREH
Iowa City, IA 52242
Phone: (319) 335-4981
Fax: (319) 335-4290
e-mail: hans-joachim-lehmler@uiowa.edu

Table of Contents

Chemicals	S3
**Table S1**. Unique chemical identifiers of the analytical standards used in this study.	S4
**Table S2.** Summary of animal body weights and tissue weights organized by sex and exposure**.**	S5
**Table S3**. Method detection limits (ng) and limits of detection (ng/g tissue) of the gas chromatographic quantification of PCB 95 and its metabolites.	S6
**Table S4**. Percent recoveries for surrogate recovery standards using liquid-liquid (LLE) or pressurized liquid extraction (PLE) for the analysis of PCB 95 and its hydroxylated metabolites in blood and tissue samples.	S7
**Table S5. Table S5.** Percent recovery of the Ongoing Precision and Recovery standards (i.e., PCB 95 and its available hydroxylated metabolites) in method blanks and tissue blanks using liquid-liquid (LLE) or pressurized liquid extraction (PLE) extraction procedures.	S8
**Table S6.** Levels of PCB 95 and its hydroxylated metabolites in adipose, blood, brain, and liver tissue (ng/g tissue).	S9
**Table S7.** Enantiomeric fractions of PCB 95 from adipose, brain, liver, and blood.	S11
**Table S8.** Statistical analysis of PCB 95 and its metabolites (ng/g tissue).	S12
**Table S9.** Statistical analysis of enantiomeric fraction (EF) values of PCB 95.	S14
**Table S10**. Similarity coefficient, cos Θ, comparing the PCB 95 metabolite profiles in adipose, blood, brain, and liver across genotypes.	S15
**Table S11.** Similarity coefficients, cos Θ, comparing the PCB 95 metabolite profiles between tissues (i.e., adipose, blood, and liver) in animals from the same genotype.	S16
**Figure S1.** Hepatic expression of transgenic CYP2A6. (A) Immunoblot detection of CYP2A6 protein in the liver.	S17
**Figure S2.** Representative chromatograms showing that the second eluting atropisomer of PCB 95 was enriched in the (a) adipose, (b) blood, (c) brain, and (d) liver of M_{WT}, $M_{KO,}$ and M_{KI} mice.	S18
**Figure S3.** Representative chromatograms showing that the second eluting atropisomer of PCB 95 was enriched in the (a) adipose, (b) blood, (c) brain, and (d) liver of F_{WT}, $F_{KO,}$ and F_{KI} mice.	S19
References	S20

Chemicals. 2,3,4',5,6-Pentachlorobiphenyl (PCB 117, PCB recovery standard), 2,2',3,4,4',5,6,6'-octachlorobiphenyl (PCB 204, internal standard) and 2,3,3',4,5,5'-hexachlorobiphenyl-4'-ol (4'-159, OH-PCB recovery standard) were purchased from Accustandard (New Haven, CT, USA). Analytical standards of hydroxylated PCB 95 metabolites, including 3-methoxy-2,2',4,5',6-pentachlorobiphenyl (methylated 2,2',4,5',6-pentachlorobiphenyl-3-ol, 3-103), 2,2',3,5',6-pentachlorobiphenyl-4-ol (4-95), 2,2',3,5',6-pentachlorobiphenyl-5-ol (5-95) and 4,5-dimethoxy-2,2',3,5',6-pentachlorobiphenyl (methylated 2,2',3,5',6-pentachlorobiphenyl-4,5-diol, 4,5-PCB 95), were synthesized as described previously.[1, 2] All the solvents, including hexane, methanol, and dichloromethane were pesticide grade level and purchased from Fisher Scientific (Waltham, MA, USA). For unique chemical identifiers of the analytical standards, see Table S1.

Table S1. Unique chemical identifiers of PCB and PCB metabolites discussed in this study.

Abbreviation	SMILES	SMARTS	StdInChI	StdInChIKey	Formula
PCB 95[a]	C1(=CC=C(C(=C1)C2=C(C(=CC=C2Cl)Cl)Cl)Cl)Cl	c1(ccc(c(c1)-c2c(c(ccc2-[Cl])-[Cl])-[Cl])-[Cl])-[Cl]	InChI=1S/C12H5Cl5/c13-6-1-2-8(14)7(5-6)11-9(15)3-4-10(16)12(11)17/h1-5H	InChIKey=GXNNLIMMEXHBKV-UHFFFAOYSA-N	C12H5Cl5
PCB 117[a]	C1=C(C=CC(=C1)C2=C(C(=CC(=C2Cl)Cl)Cl)Cl)Cl	c1c(ccc(c1)-c2c(c(cc(c2-[Cl])-[Cl])-[Cl])-[Cl])-[Cl]	InChI=1S/C12H5Cl5/c13-7-3-1-6(2-4-7)10-11(16)8(14)5-9(15)12(10)17/h1-5H	InChIKey=ZDDZPDTVCZLFFC-UHFFFAOYSA-N	C12H5Cl5
PCB 204[a]	C1=C(C=C(C(=C1Cl)C2=C(C(=C(C(=C2Cl)Cl)Cl)Cl)Cl)Cl)Cl	c1c(cc(c(c1-[Cl])-c2c(c(c(c(c2-[Cl])-[Cl])-[Cl])-[Cl])-[Cl])-[Cl])-[Cl]	InChI=1S/C12H2Cl8/c13-3-1-4(14)6(5(15)2-3)7-8(16)10(18)12(20)11(19)9(7)17/h1-2H	InChIKey=JDZUWXRNKHXZFE-UHFFFAOYSA-N	C12H2Cl8
5-95[a,b]	C1(=CC=C(C(=C1)C2=C(C(=CC(=C2Cl)O)Cl)Cl)Cl)Cl	c1(ccc(c(c1)-c2c(c(cc(c2-[Cl])-[#8])-[Cl])-[Cl])-[Cl])-[Cl]	InChI=1S/C12H5Cl5O/c13-5-1-2-7(14)6(3-5)10-11(16)8(15)4-9(18)12(10)17/h1-4,18H	InChIKey=NGZZCCQHHGJRSN-UHFFFAOYSA-N	C12H5Cl5O
4-95[a,b]	C1(=CC=C(C(=C1)C2=C(C(=C(C=C2Cl)O)Cl)Cl)Cl)Cl	c1(ccc(c(c1)-c2c(c(c(cc2-[Cl])-[#8])-[Cl])-[Cl])-[Cl])-[Cl]	InChI=1S/C12H5Cl5O/c13-5-1-2-7(14)6(3-5)10-8(15)4-9(18)11(16)12(10)17/h1-4,18H	InChIKey=VLOXUAHEXUHYTO-UHFFFAOYSA-N	C12H5Cl5O
4,5-95	C1(=CC=C(C(=C1)C2=C(C(=C(C(=C2Cl)O)O)Cl)Cl)Cl)Cl	c1(ccc(c(c1)-c2c(c(c(c(c2-[Cl])-[#8])-[#8])-[Cl])-[Cl])-[Cl])-[Cl]	InChI=1S/C12H5Cl5O2/c13-4-1-2-6(14)5(3-4)7-8(15)10(17)12(19)11(18)9(7)16/h1-3,18-19H	InChIKey=AVAUGFGSPAJGSE-UHFFFAOYSA-N	C12H5Cl5O2
Methylated 4,5-95[a]	C1(=CC=C(C(=C1)C2=C(C(=C(C(=C2Cl)OC)OC)Cl)Cl)Cl)Cl	c1(ccc(c(c1)-c2c(c(c(c(c2-[Cl])-[#8]-[#6])-[#8]-[#6])-[Cl])-[Cl])-[Cl])-[Cl]	InChI=1S/C14H9Cl5O2/c1-20-13-11(18)9(10(17)12(19)14(13)21-2)7-5-6(15)3-4-8(7)16/h3-5H,1-2H3	InChIKey=XDRJBPQGLRTGPJ-UHFFFAOYSA-N	C14H9Cl5O2
3-103[b]	C1=CC(=CC(=C1Cl)C2=C(C=C(C(=C2Cl)O)Cl)Cl)Cl	c1cc(cc(c1-[Cl])-c2c(cc(c(c2-[Cl])-[#8])-[Cl])-[Cl])-[Cl]	InChI=1S/C12H5Cl5O/c13-5-1-2-7(14)6(3-5)10-8(15)4-9(16)12(18)11(10)17/h1-4,18H	InChIKey=OTDVHFVBSACSKB-UHFFFAOYSA-N	C12H5Cl5O
Methylated 1-103[a]	C1=CC(=CC(=C1Cl)C2=C(C=C(C(=C2Cl)OC)Cl)Cl)Cl	c1cc(cc(c1-[Cl])-c2c(cc(c(c2-[Cl])-[#8]-[#6])-[Cl])-[Cl])-[Cl]	InChI=1S/C13H7Cl5O/c1-19-13-10(17)5-9(16)11(12(13)18)7-4-6(14)2-3-8(7)15/h2-5H,1H3	InChIKey=NFOJKSKYICGMGY-UHFFFAOYSA-N	C13H7Cl5O
4'-159[a]	C1(=C(C(=CC(=C1)C2=C(C(=CC(=C2Cl)Cl)Cl)Cl)Cl)O)Cl	c1(c(c(cc(c1)-c2c(c(cc(c2-[Cl])-[Cl])-[Cl])-[Cl])-[Cl])-[#8])-[Cl]	InChI=1S/C12H4Cl6O/c13-5-3-6(14)11(18)9(10(5)17)4-1-7(15)12(19)8(16)2-4/h1-3,19H	InChIKey=XWKAHVUWKLPCQI-UHFFFAOYSA-N	C12H4Cl6O

[a] Analytical standard; [b] hydroxylated PCB 95 metabolite analyzed using the corresponding methylated compounds (i.e., as a methoxylated PCB derivative).

Table S2. Summary of animal body weights and tissue weights organized by sex and exposure**.** Data are expressed as the average ± standard deviation. WT, wild-type C57BL/6 mice; KO, *Cyp2abfgs*-null mice; KI, CYP2A6-humanized mice.

**Variable**	**Male**			**Female**		
	**WT** (N=7)	**KO** (N=8)	**KI** (N=7)	**WT** (N=10)	**KO** (N=8)	**KI** (N=6)
Pre body weight (g)	30±1	44±5	40±7	23.9±3.5	31.6±4.1	28.7±5.5
Post body weight (g)	29±1	43±5	40±8	23.4±3.6	31.0±4.0	28.2±5.7
Pre BW - Post BW (g)	-0.75±0.25	-1.01±0.53	-0.51±0.32	-0.52±0.13	-0.56±0.35	-0.76±0.50
Adipose (g)#	0.68±0.18	1.33±0.19	1.22±0.47	0.43±0.31	1.17±0.31	0.95±0.50
Blood (mL)#	0.50±0.07	0.47±0.15	0.56±0.09	0.38±0.07	0.47±0.09	0.31±0.16
Brain (g)	0.34±0.03	0.34±0.03	0.33±0.01	0.35±0.02	0.36±0.05	0.33±0.03
Liver (g)	1.21±0.11	1.73±0.18†	1.67±0.43*	0.96±0.16	1.26±0.12	1.11±0.27
Brain per BW (g/g BW)	0.012±0.001	0.008±0.001†	0.009±0.002*	0.015±0.002	0.012±0.002†	0.012±0.002*
Liver per BW (g/g BW)	0.042±0.003	0.041±0.004	0.042±0.004	0.041±0.005	0.041±0.003	0.039±0.005

* Significantly different from wildtype, p-value < 0.01; † Significantly different from wildtype, p-value < 0.001.

The weight of adipose and blood was estimated based on the body weight of the mice, assuming that 5.9% and 5.85% of the body weight correspond to adipose tissue or blood, respectively.[3]

Table S3. Method detection limits (ng) and limits of quantification (ng/g tissue) of the gas chromatographic quantification of PCB 95 and its metabolites.

Analyte	Method Detection Limit[a]		Limit of Quantification[b]			
	**LLE[c]** (N=9)	**PLE[d]** (N=27)	**Adipose[d]** (N=9)	**Blood[c]** (N=6)	**Brain[d]** (N=9)	**Liver[d]** (N=10)
PCB 95	0.094	0.027	2.654	0.165	0.339	1.048
X1-95[e]	0.094	0.001	1.559	0.020	0.173	0.205
3-103	0.023	0.006	0.574	0.066	0.694	0.227
X2-95[e]	0.003	0.001	0.115	0.007	0.091	0.019
5-95	0.024	0.005	0.333	0.047	0.219	0.099
4'-95	0.040	0.009	0.857	0.085	0.222	0.169
Y1-95[e]	0.007	0.002	0.271	0.043	0.155	0.031
4-95	0.030	0.006	0.260	0.068	0.230	0.070
4,5-95	0.062	0.004	0.611	0.035	0.310	0.076
Y2-95[e]	0.004	0.001	0.048	0.005	0.047	0.048
Y3-95[e]	0.004	0.001	0.068	0.022	0.052	0.028

[a] MDL, method detection limit (ng). Calculated from the formula MDL= $mean_{blank} + t_{0.01,\ n-1} \times SD_{blank}$, where $mean_{blank}$ is the mean of blank measures, $t_{0.01,\ n-1}$ is Student's t-value for n – 1 degrees of freedom at the 99% confidence level, and SD_{blank} is the standard deviation of the blank measures.[4, 5]

[b] LOQ, (ng/g tissue) adjusted for tissue weight. Calculated from the formula LOQ = $mean_{control} + t_{0.01,\ n-1} \times SD_{control}$, where $mean_{control}$ is the mean of control tissue measures, $t_{0.01,\ n-1}$ is Student's t-value for n – 1 degrees of freedom at the 99% confidence level, and $SD_{control}$ is the standard deviation of the control tissue measures.

[c] Liquid-liquid extraction (LLE) method for blood samples.

[d] Pressurized liquid extraction (PLE) method for tissue samples.

[e] Y1-95, Y2-95, and Y3-95 are unknown di-hydroxylated metabolites. Detection limits of unknown metabolites were estimated using average relative response factors.

Table S4. Percent recoveries for surrogate recovery standards using liquid-liquid (LLE) or pressurized liquid extraction (PLE) for the analysis of PCB 95 and its hydroxylated metabolites in blood and tissue samples. Data are expressed as the average ± standard deviation (range).

**Compound**	**LLE**[a] [%] (N=61)	**PLE**[b] [%] (N=188)
PCB 117	63 ± 14 (29-91)	95 ± 13 (54-125)
4'-159	73 ± 8 (53-98)	64 ± 17 (21-104)

[a] Liquid-liquid extraction (LLE) method for blood samples.
[b] Pressurized liquid extraction (PLE) method for tissue samples.

Table S5. Percent recovery of the Ongoing Precision and Recovery standards (i.e., PCB 95 and its available hydroxylated metabolites) in method blanks and tissue blanks using liquid-liquid (LLE) or pressurized liquid extraction (PLE) extraction procedures. Data are expressed as the average ± standard deviation (range).

Compounds	PLE[a] [%]				LLE[b] [%]	
	MB (N=9)	Ad (N=3)	Br (N=3)	Li (N=3)	MB (N=3)	Bl (N=3)
PCB 117	80 ± 9 (65-92)	87 ± 3 (85-90)	96 ± 12 (82-103)	95 ± 4 (92-99)	60 ± 12 (53-74)	65 ± 7 (57-70)
4'-159	54 ± 8 (43-69)	45 ± 6 (39-50)	72 ± 7 (64-77)	68 ± 21 (44-83)	68 ± 4 (63-71)	75 ± 8 (65-81)
PCB 95	69 ± 12 (51-91)	90 ± 14 (75-102)	80 ± 11 (70-91)	97 ± 9 (89-106)	72 ± 18 (56-91)	76 ± 22 (51-91)
3-103	82 ± 9 (66-96)	98 ± 25 (78-126)	89 ± 11 (76-98)	96 ± 2 (94-98)	60 ± 7 (55-68)	63 ± 4 (59-66)
5-95	47 ± 6 (39-59)	51 ± 14 (38-66)	58 ± 11 (47-69)	63 ± 19 (43-82)	57 ± 9 (48-67)	54 ± 7 (47-60)
4'-95	85 ± 8 (70-95)	82 ± 9 (77-93)	88 ± 5 (82-93)	95 ± 8 (89-105)	62 ± 9 (57-73)	67 ± 8 (58-74)
4-95	42 ± 3 (37-48)	42 ± 2 (39-44)	43 ± 8 (35-50)	59 ± 22 (36-79)	64 ± 13 (51-77)	54 ± 12 (41-64)
4,5-95	82 ± 10 (67-96)	87 ± 3 (84-89)	91 ± 5 (88-97)	92 ± 6 (88-99)	59 ± 2 (57-60)	57 ± 19 (35-68)

[a] Liquid-liquid extraction (LLE) method for blood samples.
[b] Pressurized liquid extraction (PLE) method for tissue samples.

Table S6. Levels of PCB 95 and its hydroxylated metabolites in adipose, blood, brain, and liver tissue (ng/g tissue). Data are expressed as the mean ± standard deviation. Analytes with a grey background had a detection frequency of 100%.

**Analytes**	**M_{WT} (N=6)**	**M_{KO} (N=7)**	**M_{KI} (N=6)**	**F_{WT} (N=7)**	**F_{KO} (N=7)**	**F_{KI} (N=5)**
**Adipose**						
PCB 95	1319.1 ± 297.0 (N=6)	691.7 ± 93.0 (N=7)	733.5 ± 354.7 (N=6)	1308.3 ± 405.1 (N=7)	859.2 ± 317.4 (N=7)	824.3 ± 458.1 (N=5)
X1-95	ND	ND	ND	2.3 ± 1.1 (N=2)	ND	2.6 (N=1)
3-103	ND	1.5 ± 0.7 (N=2)	ND	0.7 (N=1)	ND	ND
X2-95	1.0 ± 0.4 (N=6)	0.5 ± 0.1 (N=7)	0.4 ± 0.3 (N=6)	0.8 ± 0.5 (N=7)	0.3 ± 0.1 (N=7)	0.7 ± 0.3 (N=5)
5-95	1.8 ± 1.0 (N=6)	3.5 ± 4.8 (N=7)	0.8 ± 0.5 (N=6)	1.3 ± 1.1 (N=7)	1.0 ± 0.4 (N=7)	1.1 ± 0.4 (N=5)
4'-95	2.1 ± 0.8 (N=6)	2.5 ± 1.7 (N=7)	1.1 ± 0.2 (N=4)	1.4 ± 0.6 (N=6)	1.5 ± 0.4 (N=6)	1.1 ± 0.3 (N=5)
Y1-95	0.7 ± 0.3 (N=6)	2.5 ± 3.5 (N=7)	0.5 ± 0.3 (N=3)	0.7 ± 0.4 (N=7)	0.6 ± 0.2 (N=7)	0.6 ± 0.3 (N=4)
4-95	0.5 ± 0.3 (N=6)	0.4 ± 0.1 (N=4)	0.3 (N=1)	0.4 ± 0.1 (N=5)	ND	ND
4,5-95	0.9 ± 0.2 (N=5)	1.9 ± 0.8 (N=7)	0.7 ± 0.2 (N=2)	0.9 ± 0.2 (N=5)	1.6 (N=1)	1.0 ± 0.6 (N=3)
Y2-95	0.06 ± 0.01 (N=3)	0.1 (N=1)	0.1 (N=1)	ND	0.1 (N=1)	ND
Y3-95	0.1 (N=1)	0.1 (N=1)	ND	ND	ND	ND
**∑OH-PCBs**	**7.0 ± 3.0 (N=6)**	**11.5 ± 11.5 (N=7)**	**2.5 ± 1.5 (N=6)**	**5.7 ± 3.7 (N=7)**	**3.4 ± 0.7 (N=7)**	**4.5 ± 1.9 (N=5)**
**Blood**						
PCB 95	14.1 ± 2.0 (N=6)	10.6 ± 7.4 (N=7)	15.6 ± 18.3 (N=6)	9.1 ± 2.7 (N=7)	8.6 ± 4.2 (N=7)	12.9 ± 15.4 (N=5)
X1-95	ND	ND	ND	ND	ND	0.2 (N=1)
3-103	0.6 ± 0.2 (N=6)	0.5 ± 0.1 (N=7)	0.4 ± 0.1 (N=6)	1.0 ± 0.5 (N=7)	0.8 ± 0.3 (N=7)	1.1 ± 1.2 (N=5)
X2-95	0.8 ± 0.2 (N=6)	0.3 ± 0.1 (N=7)	0.3 ± 0.2 (N=6)	0.5 ± 0.3 (N=7)	0.2 ± 0.1 (N=7)	1.0 ± 0.9 (N=5)
5-95	1.1 ± 0.3 (N=6)	0.7 ± 0.3 (N=7)	0.6 ± 0.3 (N=6)	0.9 ± 0.6 (N=7)	0.7 ± 0.3 (N=7)	1.9 ± 1.6 (N=5)
4'-95	3.5 ± 1.1 (N=6)	2.8 ± 0.9 (N=7)	1.3 ± 0.7 (N=6)	2.2 ± 1.0 (N=7)	2.2 ± 1.2 (N=7)	3.4 ± 2.5 (N=5)
Y1-95	26.6 ± 9.3 (N=6)	18.8 ± 4.3 (N=7)	11.9 ± 6.2 (N=6)	28.3 ± 10.6 (N=7)	18.5 ± 5.6 (N=7)	32.9 ± 27.1 (N=5)
4-95	3.2 ± 1.1 (N=6)	1.5 ± 0.8 (N=7)	1.1 ± 0.6 (N=6)	1.5 ± 0.5 (N=7)	0.4 ± 0.3 (N=7)	1.0 ± 0.8 (N=5)
4,5-95	9.1 ± 2.6 (N=6)	5.3 ± 3.8 (N=7)	3.6 ± 1.3 (N=6)	5.2 ± 1.9 (N=7)	1.4 ± 0.7 (N=7)	5.2 ± 4.8 (N=5)
Y2-95	0.02 ± 0.01 (N=6)	0.012 ± 0.004 (N=5)	0.01 (N=1)	0.02 (N=1)	0.01 (N=1)	0.01 ± 0.01 (N=3)
Y3-95	0.4 ± 0.2 (N=6)	0.4 ± 0.1 (N=7)	0.4 ± 0.3 (N=6)	0.4 ± 0.2 (N=7)	0.3 ± 0.2 (N=7)	0.7 ± 0.6 (N=5)
**∑OH-PCBs**	**45.4 ± 14.6 (N=6)**	**30.3 ± 9.0 (N=7)**	**19.6 ± 8.8 (N=6)**	**39.9 ± 15.0 (N=7)**	**24.4 ± 6.3 (N=7)**	**47.1 ± 39.2 (N=5)**

M_{WT}, male wildtype mice; M_{KO}, male *Cyp2abfgs*-null mice; M_{KI}, male CYP2A6-humanized mice; F_{WT}, female wildtype mice; F_{KO}, female *Cyp2abfgs*-null mice; F_{KI}, female CYP2A6-humanized mice. N indicates the sample number; some Ns for PCB 95 and its metabolites indicate the number of values above LOD. X1, X2, and Y1 toY3 indicate unknown mono- and di-OH PCB 95 metabolites, respectively.

Table S6--continued. Levels of PCB 95 and its hydroxylated metabolites in adipose, blood, brain, and liver tissue (ng/g tissue). Data are expressed as the mean ± standard deviation. Analytes with a grey background had a detection frequency of 100%.

Analytes	M_{WT} (N=6)	M_{KO} (N=7)	M_{KI} (N=6)	F_{WT} (N=7)	F_{KO} (N=7)	F_{KI} (N=5)
**Brain**						
PCB 95	19.3 ± 4.0 (N=6)	13.1 ± 6.1 (N=7)	13.3 ± 5.7 (N=6)	19.2 ± 7.3 (N=7)	20.6 ± 8.2 (N=7)	19.7 ± 6.8 (N=5)
X1-95	ND	ND	0.2 (N=1)	0.6 (N=1)	ND	ND
3-103	ND	ND	ND	ND	ND	ND
X2-95	ND	ND	ND	0.10 ± 0.01 (N=2)	ND	ND
5-95	ND	ND	ND	0.25 ± 0.02 (N=2)	ND	ND
4'-95	ND	0.2 (N=1)	ND	0.3 (N=1)	ND	ND
Y1-95	0.2 (N=1)	ND	ND	0.3 ± 0.1 (N=3)	0.2 ± 0.0 (N=2)	ND
4-95	ND	ND	ND	ND	ND	ND
4,5-95	ND	ND	ND	ND	ND	ND
Y2-95	ND	ND	ND	0.09 ± 0.05 (N=2)	0.07 ± 0.01 (N=2)	ND
Y3-95	0.4 ± 0.2 (N=6)	0.4 ± 0.1 (N=7)	0.4 ± 0.3 (N=6)	0.4 ± 0.2 (N=7)	0.3 ± 0.2 (N=7)	0.7 ± 0.6 (N=5)
**∑OH-PCBs**	**0.24**	**0.23**	**0.17**	**0.6 ± 0.7 (N=4)**	**0.1 ± 0.1 (N=4)**	**ND**
**Liver**						
PCB 95	96.3 ± 31.7 (N=6)	146.1 ± 42.6 (N=7)	182.6 ± 190.2 (N=6)	119.6 ± 42.1 (N=7)	108.7 ± 29.7 (N=7)	113.8 ± 44.1 (N=5)
X1-95	ND	ND	ND	ND	0.3 (N=1)	ND
3-103	0.7 ± 0.3 (N=6)	1.0 ± 0.4 (N=7)	0.6 ± 0.2 (N=6)	1.2 ± 0.4 (N=7)	1.8 ± 0.4 (N=7)	1.0 ± 0.4 (N=5)
X2-95	3.2 ± 1.6 (N=6)	3.3 ± 1.5 (N=7)	3.8 ± 2.9 (N=6)	3.6 ± 1.4 (N=7)	3.8 ± 3.9 (N=7)	5.6 ± 3.6 (N=5)
5-95	3.3 ± 1.6 (N=6)	3.7 ± 1.6 (N=7)	3.3 ± 2.1 (N=6)	3.8 ± 1.6 (N=7)	6.2 ± 1.1 (N=7)	5.5 ± 3.1 (N=5)
4'-95	5.7 ± 2.8 (N=6)	7.7 ± 3.6 (N=7)	3.8 ± 2.1 (N=6)	3.7 ± 1.8 (N=7)	8.5 ± 3.9 (N=7)	4.5 ± 2.6 (N=5)
Y1-95	4.2 ± 1.8 (N=6)	4.6 ± 1.7 (N=7)	3.3 ± 1.5 (N=6)	4.9 ± 1.7 (N=7)	8.3 ± 2.2 (N=7)	4.6 ± 2.4 (N=5)
4-95	2.0 ± 0.8 (N=6)	1.1 ± 0.9 (N=7)	0.8 ± 0.5 (N=6)	1.0 ± 0.6 (N=7)	0.4 ± 0.3 (N=7)	0.4 ± 0.1 (N=5)
4,5-95	3.4 ± 1.2 (N=6)	2.8 ± 1.6 (N=7)	1.7 ± 0.8 (N=6)	2.2 ± 0.9 (N=7)	1.2 ± 0.7 (N=7)	1.7 ± 0.3 (N=5)
Y2-95	0.07 ± 0.04 (N=3)	0.1 ± 0.1 (N=3)	0.1 ± 0.1 (N=2)	0.1 (N=1)	0.1 ± 0.1 (N=2)	ND
Y3-95	ND	0.1 ± 0.1 (N=4)	0.03 (N=1)	ND	0.2 ± 0.1 (N=5)	0.10 ± 0.02 (N=3)
**∑OH-PCBs**	**22.6 ± 9.6 (N=6)**	**24.3 ± 9.5 (N=7)**	**17.4 ± 9.3 (N=6)**	**20.4 ± 7.9 (N=7)**	**30.3 ± 5.4 (N=7)**	**23.3 ± 12.1 (N=5)**

M_{WT}, male wildtype mice; M_{KO}, male *Cyp2abfgs*-null mice; M_{KI}, male CYP2A6-humanized mice; F_{WT}, female wildtype mice; F_{KO}, female *Cyp2abfgs*-null mice; F_{KI}, female CYP2A6-humanized mice. N indicates the sample number; some Ns for PCB 95 and its metabolites indicate the number of values above LOD. X1, X2, and Y1 toY3 indicate unknown mono- and di-OH PCB 95 metabolites, respectively.

Table S7. Enantiomeric fractions of PCB 95 from adipose, brain, liver, and blood.

**Tissue**	**M_{WT}**	**M_{KO}**	**M_{KI}**	**F_{WT}**	**F_{KO}**	**F_{KI}**
Adipose	0.37 ± 0.01 (N=6)	0.38 ± 0.03 (N=7)	0.36 ± 0.02 (N=6)	0.33 ± 0.02 (N=7)	0.41 ± 0.05 (N=7)	0.35 ± 0.02 (N=5)
Blood	0.28 ± 0.02 (N=6)	0.35 ± 0.04 (N=7)	0.31 ± 0.03 (N=6)	0.32 ± 0.07 (N=6)	0.37 ± 0.07 (N=7)	0.28 ± 0.07 (N=4)
Brain	0.31 ± 0.02 (N=6)	0.34 ± 0.03 (N=7)	0.32 ± 0.02 (N=5)	0.25 ± 0.03 (N=7)	0.39 ± 0.05 (N=7)	0.29 ± 0.01 (N=3)
Liver	0.26 ± 0.02 (N=6)	0.28 ± 0.04 (N=7)	0.27 ± 0.03 (N=6)	0.20 ± 0.03 (N=7)	0.32 ± 0.06 (N=7)	0.24 ± 0.03 (N=5)

M_{WT}, male wildtype mice; M_{KO}, male *Cyp2abfgs*-null mice; M_{KI}, male CYP2A6-humanized mice; F_{WT}, female wildtype mice; F_{KO}, female *Cyp2abfgs*-null mice; F_{KI}, female CYP2A6-humanized mice.

Table S8. P-values from the statistical analysis comparing the levels of PCB 95 and its metabolites (ng/g tissue). Statistical analyses were performed by two-way ANOVA analysis tool with Bonferroni correction for multiple comparisons in GraphPad Prism 9.4.1; p-values < 0.05 are indicated by a grey background.

Analytes	tissue	M_{WT}-M_{KO}	M_{WT}-M_{KI}	M_{KO}-M_{KI}	F_{WT}-F_{KO}	F_{WT}-F_{KI}	F_{KO}-F_{KI}	M_{WT}-F_{WT}	M_{KO}-F_{KO}	M_{KI}-F_{KI}
PCB 95	Ad	<0.0001	<0.0001	>0.9999	<0.0001	<0.0001	>0.9999	0.8465	>0.9999	<0.0001
	Bl	>0.9999	>0.9999	>0.9999	>0.9999	>0.9999	>0.9999	>0.9999	>0.9999	>0.9999
	Br	>0.9999	>0.9999	>0.9999	>0.9999	>0.9999	>0.9999	>0.9999	>0.9999	>0.9999
	Li	>0.9999	>0.9999	>0.9999	>0.9999	>0.9999	>0.9999	>0.9999	>0.9999	>0.9999
X1-95	Ad	>0.9999	>0.9999	>0.9999	>0.9999	>0.9999	>0.9999	>0.9999	>0.9999	>0.9999
	Bl	>0.9999	>0.9999	>0.9999	>0.9999	>0.9999	>0.9999	>0.9999	>0.9999	>0.9999
	Br	>0.9999	>0.9999	0.0007	>0.9999	>0.9999	>0.9999	0.0004	0.0017	>0.9999
	Li	>0.9999	>0.9999	>0.9999	>0.9999	>0.9999	>0.9999	>0.9999	>0.9999	>0.9999
3-103	Ad	>0.9999	>0.9999	>0.9999	>0.9999	>0.9999	>0.9999	>0.9999	>0.9999	>0.9999
	Bl	>0.9999	>0.9999	>0.9999	>0.9999	>0.9999	>0.9999	>0.9999	>0.9999	>0.9999
	Br	>0.9999	>0.9999	>0.9999	>0.9999	>0.9999	>0.9999	>0.9999	>0.9999	>0.9999
	Li	>0.9999	>0.9999	>0.9999	>0.9999	>0.9999	>0.9999	>0.9999	>0.9999	>0.9999
X2-95	Ad	>0.9999	>0.9999	>0.9999	>0.9999	>0.9999	>0.9999	>0.9999	>0.9999	>0.9999
	Bl	>0.9999	>0.9999	>0.9999	>0.9999	>0.9999	>0.9999	>0.9999	>0.9999	>0.9999
	Br	>0.9999	>0.9999	>0.9999	>0.9999	>0.9999	>0.9999	>0.9999	>0.9999	>0.9999
	Li	>0.9999	>0.9999	>0.9999	>0.9999	>0.9999	0.9813	>0.9999	0.5125	0.8226
5-95	Ad	0.0204	0.8744	>0.9999	<0.0001	<0.0001	>0.9999	>0.9999	>0.9999	>0.9999
	Bl	>0.9999	>0.9999	>0.9999	>0.9999	>0.9999	>0.9999	>0.9999	>0.9999	>0.9999
	Br	>0.9999	>0.9999	>0.9999	>0.9999	>0.9999	>0.9999	>0.9999	>0.9999	>0.9999
	Li	>0.9999	>0.9999	>0.9999	>0.9999	0.045	0.2952	0.0662	>0.9999	>0.9999
4'-95	Ad	>0.9999	0.4699	>0.9999	0.0452	0.3343	>0.9999	>0.9999	>0.9999	>0.9999
	Bl	>0.9999	>0.9999	>0.9999	>0.9999	>0.9999	>0.9999	>0.9999	>0.9999	>0.9999
	Br	>0.9999	>0.9999	>0.9999	>0.9999	>0.9999	>0.9999	>0.9999	>0.9999	>0.9999
	Li	0.3478	0.542	0.3463	0.0002	>0.9999	>0.9999	<0.0001	>0.9999	0.0002

M_{WT}, male wildtype mice; M_{KO}, male *Cyp2abfgs*-null mice; M_{KI}, male CYP2A6-humanized mice; F_{WT}, female wildtype mice; F_{KO}, female *Cyp2abfgs*-null mice; F_{KI}, female CYP2A6-humanized mice; Ad, adipose tissue; Bl, blood; Br, brain; Li, liver.

Table S8--continued. P-values from the statistical analysis comparing the levels of PCB 95 and its metabolites (ng/g tissue). Statistical analyses were performed by two-way ANOVA analysis tool with Bonferroni correction for multiple comparisons in GraphPad Prism 9.4.1; p-values < 0.05 are indicated by a grey background.

Analytes	tissue	M_{WT}-M_{KO}	M_{WT}-M_{KI}	M_{KO}-M_{KI}	F_{WT}-F_{KO}	F_{WT}-F_{KI}	F_{KO}-F_{KI}	M_{WT}-F_{WT}	M_{KO}-F_{KO}	M_{KI}-F_{KI}
	Ad	0.0092	>0.9999	>0.9999	0.0004	0.0019	>0.9999	>0.9999	>0.9999	>0.9999
	Bl	0.0051	<0.0001	>0.9999	0.0251	>0.9999	<0.0001	<0.0001	0.7274	<0.0001
	Br	>0.9999	>0.9999	0.4356	>0.9999	>0.9999	>0.9999	0.7694	0.0471	>0.9999
4-95	Li	>0.9999	>0.9999	>0.9999	>0.9999	0.0002	>0.9999	0.0009	>0.9999	0.0009
	Ad	>0.9999	>0.9999	>0.9999	>0.9999	>0.9999	>0.9999	>0.9999	>0.9999	>0.9999
	Bl	>0.9999	>0.9999	>0.9999	>0.9999	>0.9999	>0.9999	>0.9999	>0.9999	>0.9999
	Br	>0.9999	>0.9999	>0.9999	>0.9999	>0.9999	>0.9999	>0.9999	>0.9999	>0.9999
Y1-95	Li	>0.9999	>0.9999	>0.9999	>0.9999	>0.9999	>0.9999	>0.9999	>0.9999	>0.9999
	Ad	0.8055	>0.9999	>0.9999	0.1476	0.1575	>0.9999	>0.9999	>0.9999	>0.9999
	Bl	>0.9999	0.2322	>0.9999	>0.9999	0.9183	>0.9999	>0.9999	>0.9999	>0.9999
	Br	>0.9999	>0.9999	>0.9999	>0.9999	>0.9999	>0.9999	>0.9999	>0.9999	>0.9999
4,5-95	Li	>0.9999	0.9926	>0.9999	>0.9999	0.6821	>0.9999	>0.9999	>0.9999	>0.9999
	Ad	>0.9999	>0.9999	>0.9999	>0.9999	>0.9999	>0.9999	>0.9999	>0.9999	>0.9999
	Bl	>0.9999	>0.9999	>0.9999	>0.9999	>0.9999	>0.9999	>0.9999	>0.9999	>0.9999
	Br	>0.9999	>0.9999	>0.9999	>0.9999	>0.9999	>0.9999	>0.9999	>0.9999	>0.9999
Y2-95	Li	>0.9999	>0.9999	>0.9999	>0.9999	>0.9999	>0.9999	>0.9999	>0.9999	>0.9999
	Ad	>0.9999	>0.9999	>0.9999	>0.9999	>0.9999	>0.9999	>0.9999	>0.9999	>0.9999
	Bl	>0.9999	>0.9999	>0.9999	>0.9999	>0.9999	>0.9999	>0.9999	>0.9999	>0.9999
	Br	>0.9999	>0.9999	>0.9999	>0.9999	>0.9999	>0.9999	>0.9999	>0.9999	>0.9999
Y3-95	Li	>0.9999	>0.9999	>0.9999	>0.9999	>0.9999	>0.9999	>0.9999	>0.9999	>0.9999
	Ad	0.5191	0.0001	0.1327	0.7907	0.9997	0.2468	0.1948	0.1735	0.7240
	Bl	0.0013	<0.0001	0.0002	<0.0001	0.9975	0.0928	0.7252	0.1358	0.0027
	Br	0.3388	0.3739	0.4142	0.6706	0.7583	0.1547	0.7593	0.0515	0.6009
∑OH-PCBs*	Li	<0.0001	<0.0001	0.5778	0.8345	<0.0001	<0.0001	<0.0001	0.1091	0.0076

M_{WT}, male wildtype mice; M_{KO}, male *Cyp2abfgs*-null mice; M_{KI}, male CYP2A6-humanized mice; F_{WT}, female wildtype mice; F_{KO}, female *Cyp2abfgs*-null mice; F_{KI}, female CYP2A6-humanized mice; Ad, adipose tissue; Bl, blood; Br, brain; Li, liver. * interaction between metabolite factor and genotype factor.

Table S9. P-values from the statistical analysis of enantiomeric fraction (EF) values of PCB 95. Statistical analyses were performed by two-way ANOVA analysis tool with Bonferroni correction for multiple comparisons in GraphPad Prism 9.4.1; p-values < 0.05 are indicated by a grey background.

**Tissue**	M_{WT}-M_{KO}	M_{WT}-M_{KI}	M_{KO}-M_{KI}	F_{WT}-F_{KO}	F_{WT}-F_{KI}	F_{KO}-F_{KI}	M_{WT}-F_{WT}	M_{KO}-F_{KO}	M_{KI}-F_{KI}
Adipose	>0.9999	>0.9999	>0.9999	0.0042	>0.9999	0.1238	>0.9999	>0.9999	>0.9999
Blood	0.057	>0.9999	>0.9999	0.342	>0.9999	0.0031	>0.9999	>0.9999	>0.9999
Brain	>0.9999	>0.9999	>0.9999	<0.0001	>0.9999	0.0157	0.1107	0.3873	>0.9999
Liver	>0.9999	>0.9999	>0.9999	<0.0001	>0.9999	0.0293	0.1463	>0.9999	>0.9999

M_{WT}, male wildtype mice; M_{KO}, male *Cyp2abfgs*-null mice; M_{KI}, male CYP2A6-humanized mice; F_{WT}, female wildtype mice; F_{KO}, female *Cyp2abfgs*-null mice; F_{KI}, female CYP2A6-humanized mice; Ad, adipose tissue; Bl, blood; Br, brain; Li, liver.

Table S10. Similarity coefficient, cos Θ, comparing the PCB 95 metabolite profiles in adipose, blood, brain, and liver across genotypes.

**Mouse model**	**M_{WT}**	**M_{KO}**	**M_{KI}**	**F_{WT}**	**F_{KO}**	**F_{KI}**
**Adipose**						
M_{WT}	1.00	0.88	0.98	0.70	0.89	0.60
M_{KO}		1.00	0.89	0.69	0.85	0.56
M_{KI}			1.00	0.70	0.95	0.61
F_{WT}				1.00	0.66	0.96
F_{KO}					1.00	0.60
F_{KI}						1.00
**Blood**						
M_{WT}	1.00	1.00	1.00	0.99	0.96	0.98
M_{KO}		1.00	1.00	0.99	0.98	0.99
M_{KI}			1.00	0.99	0.97	0.99
F_{WT}				1.00	0.99	1.00
F_{KO}					1.00	1.00
F_{KI}						1.00
**Brain**						
M_{WT}	1.00	0.75	0.78	0.56	0.99	0.87
M_{KO}		1.00	0.78	0.55	0.72	0.86
M_{KI}			1.00	0.71	0.75	0.90
F_{WT}				1.00	0.57	0.46
F_{KO}					1.00	0.83
F_{KI}						1.00
**Liver**						
M_{WT}	1.00	0.98	0.96	0.96	0.93	0.92
M_{KO}		1.00	0.95	0.94	0.96	0.91
M_{KI}			1.00	0.98	0.94	0.99
F_{WT}				1.00	0.96	0.97
F_{KO}					1.00	0.93
F_{KI}						1.00

The similarity coefficient, cos Θ, indicates the similarity of two profiles, with values between 1 and 0.[6] A value of 1 indicates that two data sets are identical. A value of 0 indicates that the data sets are completely different. M_{WT}, male wildtype mice; M_{KO}, male *Cyp2abfgs*-null mice; M_{KI}, male CYP2A6-humanized mice; F_{WT}, female wildtype mice; F_{KO}, female *Cyp2abfgs*-null mice; F_{KI}, female CYP2A6-humanized mice.

Table S11. Similarity coefficients, cos Θ, comparing the PCB 95 metabolite profiles between tissues (i.e., adipose, blood, and liver) in animals from the same genotype.

Tissue	Adipose	Blood	Liver
**M_{WT}**			
Adipose	1.00	0.43	0.95
Blood		1.00	0.66
Liver			1.00
**M_{KO}**			
Adipose	1.00	0.62	0.88
Blood		1.00	0.60
Liver			1.00
**M_{KI}**			
Adipose	1.00	0.51	0.92
Blood		1.00	0.59
Liver			1.00
**F_{WT}**			
Adipose	1.00	0.32	0.69
Blood		1.00	0.67
Liver			1.00
**F_{KO}**			
Adipose	1.00	0.38	0.77
Blood		1.00	0.68
Liver			1.00
**F_{KI}**			
Adipose	1.00	0.29	0.58
Blood		1.00	0.55
Liver			1.00

The similarity coefficient, cos Θ, indicates the similarity of two profiles, with values between 1 and 0.[6] A value of 1 indicates that two profiles are identical. A value of 0 indicates that the profiles are completely different. The brain tissue was not included here because only a few metabolites were detected in this tissue. M_{WT}, male wildtype mice; M_{KO}, male *Cyp2abfgs*-null mice; M_{KI}, male CYP2A6-humanized mice; F_{WT}, female wildtype mice; F_{KO}, female *Cyp2abfgs*-null mice; F_{KI}, female CYP2A6-humanized mice.

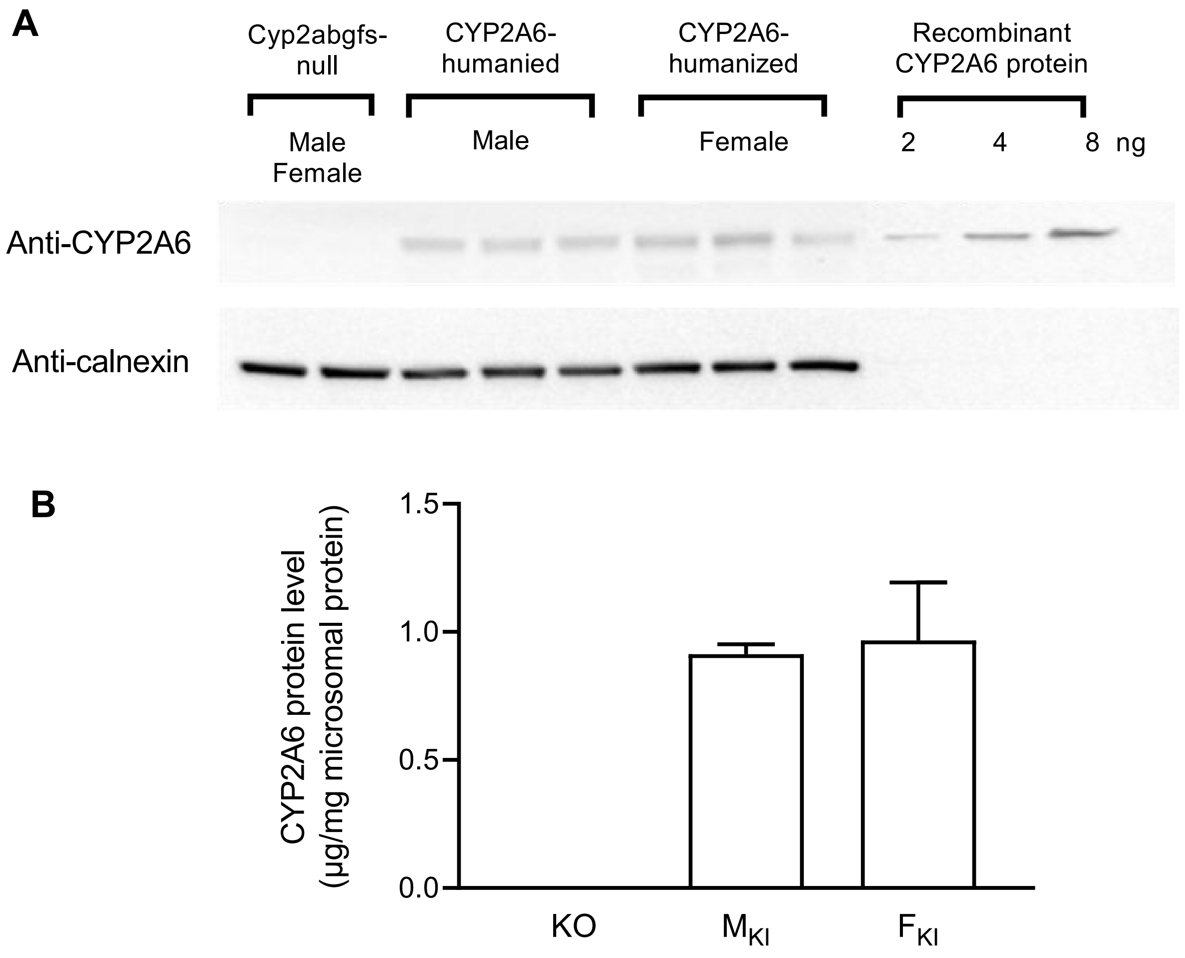

Figure S1. Hepatic expression of transgenic CYP2A6. (A) Immunoblot detection of CYP2A6 protein in the liver from individual mice. Microsomal samples (10 µg protein per lane) prepared from livers of 2-month-old male and female *Cyp2abfgs*-null or CYP2A6-humanized mice (hemizygous, n = 3) were analyzed with an anti-CYP2A6 antibody. Recombinant CYP2A6 standards (2, 4, or 8 ng protein) were used for quantitative analysis. (B) Results of densitometric analysis of immunoblot data for both male and female mice (n = 3). There was no significant sex difference in CYP2A6 protein levels (p>0.05, t-test). For details regarding the immunoblot analysis, see Materials and Methods. KO, *Cyp2abfgs*-null mice; M_{KI} and MF_{KI}, male and female CYP2A6-humanized mice, respectively.

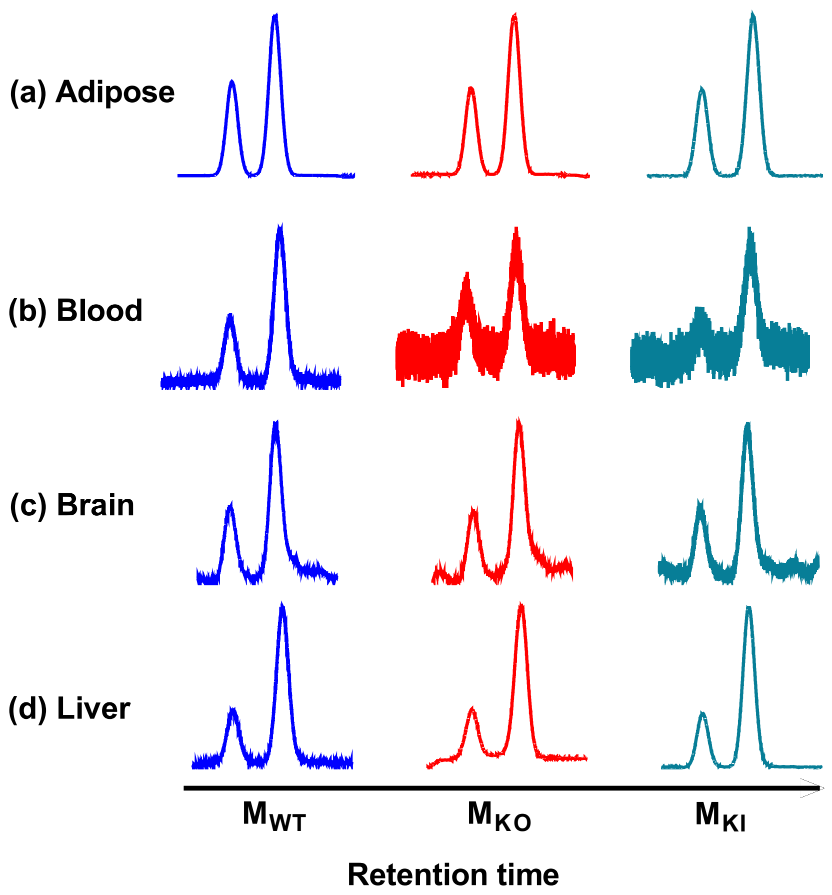

Figure S2. Representative chromatograms showing that the second eluting atropisomer of PCB 95 was enriched in the (a) adipose, (b) blood, (c) brain, and (d) liver of M_{WT}, M_{KO}, and M_{KI} mice. The enantioselective analyses were performed on a Chirasil Dex CB capillary column as described under Materials and Methods. The PCB 95 atropisomer eluting first (E1-PCB 95) and second (E2-PCB 95) correspond to aR- and aS-PCB 95, respectively.[7, 8] M_{WT}, male wildtype mice; M_{KO}, male *Cyp2abfgs*-null mice; M_{KI}, male CYP2A6-humanized mice; F_{WT}, female wildtype mice; F_{KO}, female *Cyp2abfgs*-null mice; F_{KI}, female CYP2A6-humanized mice.

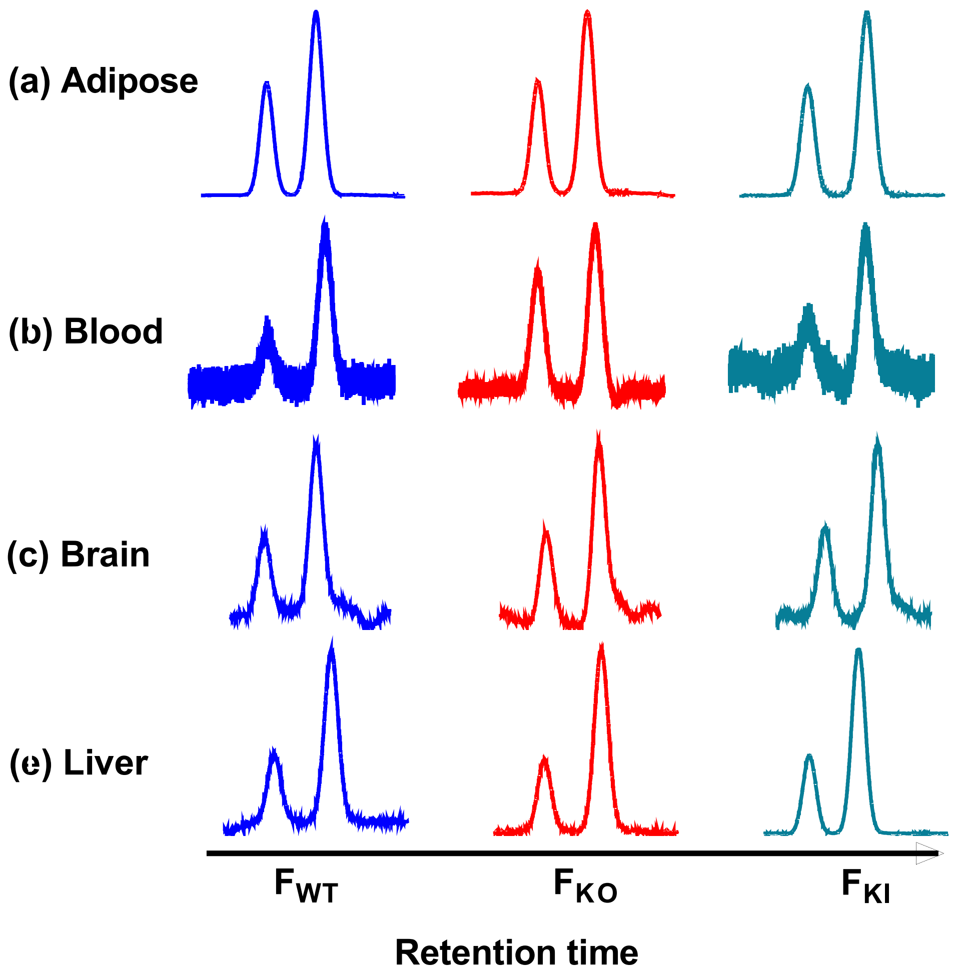

Figure S3. Representative chromatograms showing that the second eluting atropisomer of PCB 95 was enriched in the (a) adipose, (b) blood, (c) brain, and (d) liver of F_{WT}, $F_{KO,}$ and F_{KI} mice. The enantioselective analyses were performed on a Chirasil Dex CB capillary column as described under Materials and Methods. The PCB 95 atropisomer eluting first (E1-PCB 95) and second (E2-PCB 95) correspond to aR- and aS-PCB 95, respectively.[7, 8] M_{WT}, male wildtype mice; M_{KO}, male Cyp2abfgs-null mice; M_{KI}, male CYP2A6-humanized mice; F_{WT}, female wildtype mice; F_{KO}, female Cyp2abfgs-null mice; F_{KI}, female CYP2A6-humanized mice.

References

1. Kania-Korwel, I.; Vyas, S. M.; Song, Y.; Lehmler, H.-J., Gas chromatographic separation of methoxylated polychlorinated biphenyl atropisomers. *J. Chromatogr. A* **2008,** *1207*, (1-2), 146-154.
2. Joshi, S. N.; Vyas, S. M.; Duffel, M. W.; Parkin, S.; Lehmler, H.-J., Synthesis of sterically hindered polychlorinated biphenyl derivatives. *Synthesis* **2011**, (7), 1045-1054.
3. Kania-Korwel, I.; Shaikh, N. S.; Hornbuckle, K. C.; Robertson, L. W.; Lehmler, H.-J., Enantioselective disposition of PCB 136 (2,2',3,3',6,6'-hexachlorobiphenyl) in C57BL/6 mice after oral and intraperitoneal administration. *Chirality* **2007,** *19*, (1), 56-66.
4. Hu, D. F.; Lehmler, H. J.; Martinez, A.; Wang, K.; Hornbuckle, K. C., Atmospheric PCB congeners across Chicago. *Atmos. Environ.* **2010,** *44*, (12), 1550-1557.
5. United States Environmental Protection Agency, Clean Water Act Analytical methods. Method Detection Limit - Frequent Questions. *https://www.epa.gov/cwa-methods/method-detection-limit-frequent-questions* (accessed June 19, 2023).
6. Davis, J. C., *Statistics and Data Analysis in Geology*. Wiley: New York, 1986.
7. Feng, W.; Zheng, J.; Robin, G.; Dong, Y.; Ichikawa, M.; Inoue, Y.; Mori, T.; Nakano, T.; Pessah, I. N., Enantioselectivity of 2,2',3,5',6-pentachlorobiphenyl (PCB 95) atropisomers toward ryanodine receptors (RyRs) and their influences on hippocampal neuronal networks. *Environ. Sci. Technol.* **2017,** *51*, (24), 14406-14416.
8. Nagayoshi, H.; Kakimoto, K.; Konishi, Y.; Kajimura, K.; Nakano, T., Determination of the human cytochrome P450 monooxygenase catalyzing the enantioselective oxidation of 2,2',3,5',6-pentachlorobiphenyl (PCB 95) and 2,2',3,4,4',5',6-heptachlorobiphenyl (PCB 183). *Environ. Sci. Poll. Res.* **2018**, *25*, (17), 16420-16426.
